# Visible-Spectrum Proxy and Texture Features Coupled with Optimized Regression for Predicting Key Minerals in Chinese Wolfberry

**DOI:** 10.3390/foods15162786

**Published:** 2026-08-08

**Authors:** Peng Chen, Rao Fu, Linjing Zhu, Ke Zhang, Xialin Chen, Yuwen Zhao, Peina Zhou, Chenghao Fei

**Affiliations:** 1 College of Horticulture, Nanjing Agricultural University, Nanjing 211800, China; chenpenggzy@163.com (P.C.); fur@stu.njau.edu.cn (R.F.); 2024104134@stu.njau.edu.cn (L.Z.); zhangk@njau.edu.cn (K.Z.); 2 State Key Laboratory on Technologies for Chinese Medicine Pharmaceutical Process Control and Intelligent Manufacture, Nanjing 210023, Chinadundunerr@163.com (Y.Z.); 3 Institute of Plant Resources and Chemistry, Nanjing Research Institute for Comprehensive Utilization of Wild Plants, Nanjing 210042, China; 4 State Key Laboratory for Quality Ensurance and Sustainable Use of Dao-di Herbs, Beijing 100700, China

**Keywords:** Chinese wolfberry, RGB image analysis, visible-color spectral proxy, frequency-domain texture, mineral elements, DBO-RBF

## Abstract

Chinese wolfberry (CW) is a medicinal-food homologous fruit. Key mineral elements are important indicators of the nutritional quality and geographical authenticity of CW. Rapid and non-destructive evaluation of key mineral elements remains challenging because inductively coupled plasma mass spectrometry (ICP-MS) is accurate but destructive and laboratory-dependent. In this study, 120 CW batches from Ningxia, Qinghai, Gansu, and Xinjiang were analyzed by combining ICP-MS reference measurements of Cu, Fe, Mn, and Zn with standardized RGB image features. Visible-color proxy curves were reconstructed from RGB/L*a*b* information and Gaussian fitting, and frequency-domain texture descriptors were extracted from Fourier-derived angle–energy curves. Multivariate analysis showed that mineral profiles, the 570–593 nm visible-color proxy band, and the 0–40° and 63–140° texture-angle ranges contributed to regional differentiation. A Dung Beetle Optimizer-radial basis function (DBO-RBF) regression model was then used for internal prediction of mineral element contents and compared with nine baseline regression algorithms. DBO-RBF achieved test-set R^2^ values of 0.949, 0.965, 0.951, and 0.913 for Cu, Fe, Mn, and Zn, respectively, with corresponding test RPD values of 2.9017, 3.2419, 3.0175, and 2.8416. These results indicate that image-derived visible-color and texture features can provide useful screening information for mineral quality assessment in CW, offering a rapid, low-cost, and non-destructive approach for preliminary batch evaluation.

## 1. Introduction

Chinese wolfberry (CW, also widely known as gouqizi) is the dried mature fruit of *Lycium barbarum* L., a plant species within the Lycium genus of the Solanaceae family [1,2]. They are the dried mature fruits of Ningxia goji plants and represent a traditional and classic medicinal-food dual-use resource in China. The primary production of CW is concentrated in the arid regions of northwest China, particularly in Zhongning, Ningxia, known for its superior quality [3,4]. Published estimates place the annual production of dried goji fruit in China’s principal growing regions at approximately 25,000–30,000 metric tons [5]. This variety is the only one listed in the Chinese Pharmacopoeia [6,7]. CW is rich in bioactive nutrients like polysaccharides, amino acids, flavonoids, carotenoids, and trace minerals such as Cu, Fe, Mn, and Zn, which form its core nutritional profile [8]. CW offers antioxidant benefits, immune support, liver protection, and gut health improvement, aligning with modern preventive healthcare trends [9]. As a result, CW is widely used in health supplements, pharmaceuticals, and diets, with growing market demand.

The nutritional quality and market value of CW are primarily determined by their intrinsic composition, including nutrients like polysaccharides, amino acids, and essential trace minerals such as Cu, Fe, Mn, and Zn. These elements dictate their nutritional and application value [10,11,12,13]. Published mineral concentrations vary with cultivar, geographical origin, sample preparation, and moisture basis. In fresh red and yellow L. barbarum fruits, Cu, Fe, Mn, and Zn concentrations were 0.39–0.48, 1.15–2.20, 0.22–0.25, and 0.91–0.97 mg/100 g fresh weight, respectively [14]. A survey of 185 oven-dried Ningxia wolfberry samples reported mean Cu and Zn concentrations of 8.70 ± 2.70 and 19.6 ± 6.41 mg/kg, respectively, with corresponding ranges of 2.29–14.5 and 11.0–35.4 mg/kg [15]. Cu, Fe, Mn, and Zn were selected because they contribute to oxygen transport and energy metabolism, iron utilization and antioxidant defense, enzymatic metabolism, and immune and protein synthesis, respectively [10,11,12,13]. Their contents vary with origin, soil, and cultivation conditions, making them useful indicators of CW mineral quality. This study therefore focused on these four nutritionally relevant elements rather than a comprehensive mineral profile. Regional factors like soil, climate, and cultivation methods cause significant variations in CW’s composition, especially in mineral content, affecting their quality and market value [16]. CW from genuine production areas is more valuable due to its superior elemental composition and nutritional balance. Accurate detection of key mineral elements in CW is crucial for assessing nutritional quality and grading [17,18]. Current methods have significant limitations: ICP-MS is precise but complex, slow, and destructive, while manual evaluations are subjective and inaccurate for internal indicators [19]. Thus, developing a precise, efficient, and non-destructive detection system for CW is urgently needed to advance the industry.

Non-destructive testing has become an important direction in agricultural product quality evaluation. At present, visible/near-infrared spectroscopy, hyperspectral imaging, X-ray fluorescence, laser-induced breakdown spectroscopy, ICP-MS-based elemental fingerprinting, stable-isotope analysis and RGB/computer-vision approaches have been used for food composition prediction, origin authentication, and quality control [1,2,20,21,22,23,24]. These techniques provide different types of evidence. Spectroscopic and elemental methods usually have stronger chemical specificity, but they often require specialized instruments and higher analytical costs. RGB imaging, in contrast, is low-cost, rapid, and easy to deploy, although its features mainly reflect external color and texture phenotypes rather than direct molecular or elemental signals. Therefore, RGB-derived descriptors are more appropriate as a front-end screening tool. If standardized color and texture features are correlated with mineral variation, they may provide useful preliminary information for CW quality evaluation.

In this study, 120 CW batches from Ningxia (NX), Qinghai (QH), Gansu (GS), and Xinjiang (XJ) were analyzed. Standardized RGB images were used to extract visible-color proxy features and Fourier-derived angular texture features, while ICP-MS was used to determine Cu, Fe, Mn, and Zn contents. Principal component analysis (PCA), partial least squares discriminant analysis (PLS-DA), and variable importance in projection (VIP) analysis were applied to identify image-derived features associated with mineral profiles. A Dung Beetle Optimizer-radial basis function (DBO-RBF) regression model and comparative regression algorithms were then used to evaluate the internal predictive ability of these features for key mineral elements. Three a priori hypotheses were formulated: (H1) Cu, Fe, Mn, and Zn concentrations differ among CW samples from the four production regions; (H2) regional mineral variation is reflected in standardized RGB color and Fourier-derived texture features; and (H3) VIP-selected image features combined with nonlinear DBO-RBF regression yield higher predictive accuracy for mineral concentrations than the comparative regression algorithms. This work aimed to establish a low-cost image-based screening strategy for CW quality assessment, providing a rapid, non-destructive, and easily deployable approach for preliminary mineral element evaluation, batch-level quality grading, and high-throughput sample screening.

## 2. Materials and Methods

### 2.1. Sample Collection

**Figure 1:**
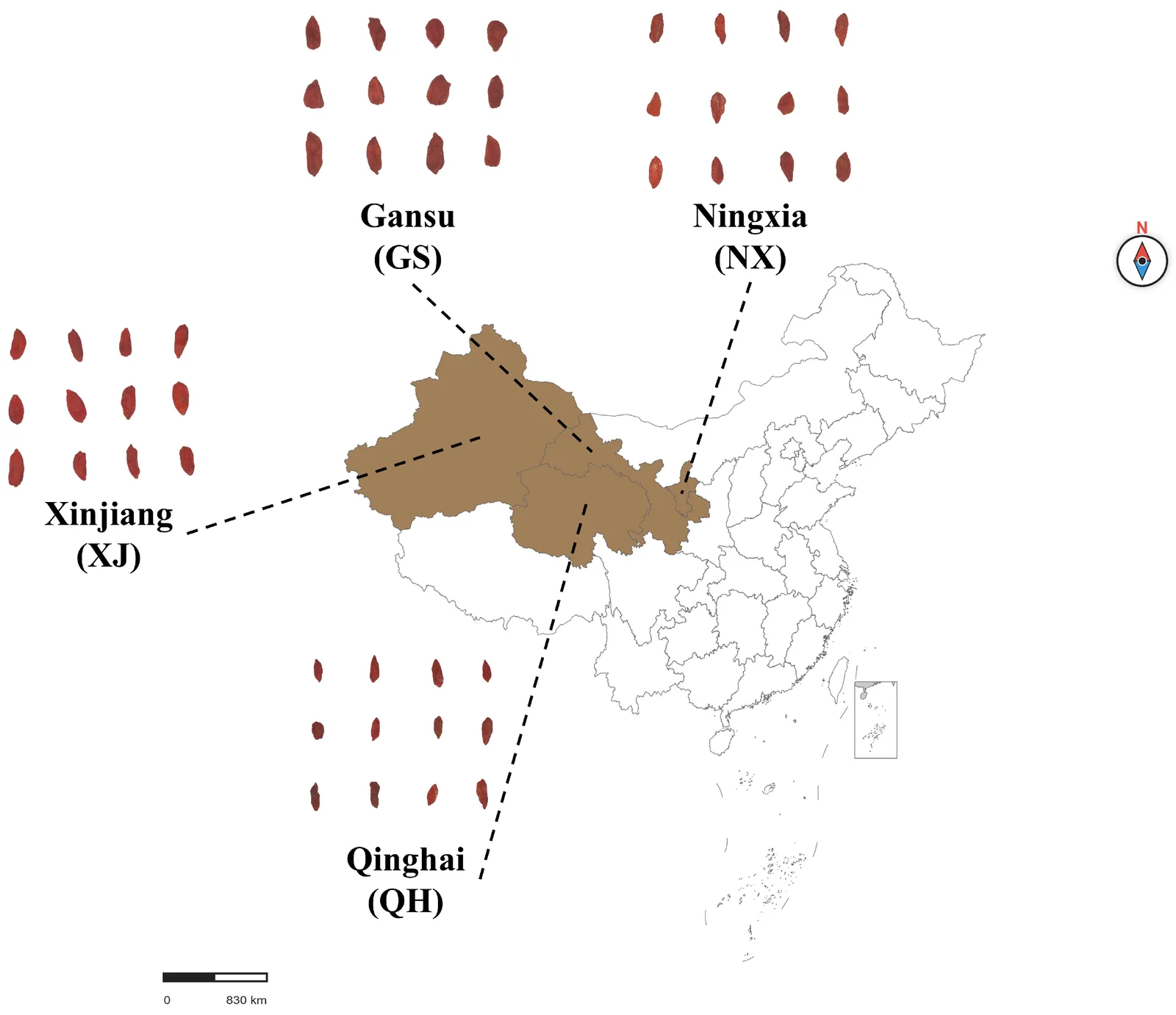
Geographic information for the CW samples. QH, Qinghai; XJ, Xinjiang; NX, Ningxia; GS, Gansu.

This study acquired a total of 120 batches of CW samples from the four principal production regions of Xinjiang, Gansu, Ningxia, and Qinghai. These regions were selected because they represent the principal commercial production areas of CW in China and encompass substantial geographical and environmental variation. Ningxia includes the traditional production area recognized for CW cultivation, whereas Xinjiang, Gansu, and Qinghai represent major production regions with distinct climatic and ecological conditions. The inclusion of these four regions therefore provided representative samples for evaluating geographical differences in mineral composition and image-derived characteristics. An equal number of samples, specifically 30 batches, were collected from each region to ensure a uniform distribution. Within each region, samples were obtained from independent production lots. Mature and intact dried fruits without visible mildew, insect damage, or abnormal discoloration were selected. Each batch was placed in a sealed food-grade polyethylene bag and protected from moisture, direct light, and mechanical damage during transportation. The samples were transported to the laboratory under dry ambient conditions and registered immediately upon arrival. The geographical range of collection points spanned 31.36° N to 48.10° N latitude and 73.40° E to 108.46° E longitude. Post-collection, all samples were subjected to a standardized pretreatment process, which included rinsing with deionized water to remove impurities, drying at 45 °C for 12 h to eliminate residual moisture and prevent interference with subsequent analyses, and storage at −20 °C to preserve sample quality and stability prior to analysis. For accurate management and traceability, each sample batch received a unique identifier, combining “CW” (for CW) with a regional code (CW-XJ for Xinjiang, CW-GS for Gansu, CW-NX for Ningxia, CW-QH for Qinghai) to indicate variety and origin. Detailed maps and metadata for sample origins are provided in Figure 1 and Table S1.

### 2.2. Computer-Vision-Derived Visible-Color and Frequency-Domain Feature Extraction

#### 2.2.1. Standardized Sample Image Acquisition

To minimize environmental variability during image-derived feature extraction, a standardized and repeatable RGB image-acquisition system was established. The system comprised an industrial RGB camera (MV-CA020-20GC; Hikrobot, Hangzhou, China) with an image-sensor resolution of 1920 × 1080 pixels, a ring-shaped LED illumination source with a correlated color temperature of 5500 K, and a matte white, non-reflective sample stage. The illuminance uniformity at the sample plane was maintained at no less than 90%. The camera, illumination source, and sample stage were fixed in position throughout image acquisition to maintain constant imaging geometry and illumination conditions. All preprocessed CW samples were evenly spread as a single layer over the central area of the sample stage at a consistent thickness. This procedure minimized image variation associated with particle overlap, uneven sample coverage, background reflection, and local shadowing. The camera optical axis was aligned perpendicular to the sample stage, and the camera-to-sample distance was fixed at 30 cm. Images were acquired using fixed camera settings, including an exposure time of 1/200 s and an ISO sensitivity of 100. Image resolution, exposure time, ISO sensitivity, camera position, lighting configuration, and background conditions were kept unchanged for all batches. For each batch, three images were acquired at predefined sample orientations under the same acquisition settings. Each image was inspected for focus, illumination uniformity, glare, shadowing, and sample occlusion. The image with the best sharpness and without visible glare, shadowing, or obstruction was retained as the representative RGB image for subsequent color and texture feature extraction.

#### 2.2.2. Visible-Color Spectral Proxy Reconstruction and Feature Parameter Extraction

Visible-color proxy features were reconstructed from standardized RGB images through sample-region segmentation, color-space conversion, and Gaussian curve fitting [25,26]. The effective CW region was first extracted after excluding the reference color card and background area. The mean red, green, and blue channel values of each sample region were calculated and corrected using a gamma value of 2.2. The corrected RGB values were converted to CIE XYZ tristimulus values and then transformed into CIE L*a*b* color coordinates.

Based on the L*a*b*-derived weighting rules, Gaussian parameters were assigned to seven nominal visible-color bands. These Gaussian functions were superimposed at 1 nm intervals across 380–780 nm to generate a continuous visible-color proxy curve. The reconstructed curve was used to describe the image-derived color distribution in the visible range. It represents an optical phenotype extracted from RGB images, rather than directly measured reflectance, absorbance, or multispectral information. Therefore, band-level differences were interpreted as color-related phenotypic variation associated with CW quality characteristics. Detailed algorithmic steps are provided in Table S2.

#### 2.2.3. Energy Spectrum Feature Extraction and Quantification

Frequency-domain texture features were extracted from grayscale CW images using Fourier transform (FT) analysis [27,28]. This conversion isolated spatial intensity variation from color information. After spectrum centering, the resulting Fourier spectrum was represented as a frequency-energy map and analyzed in polar coordinates. Radial frequency energy was integrated across angular bins from 0° to 180° to generate angle–energy curves describing the directional distribution of image texture. Peak intensity, peak angle, curve width, and integrated energy were then calculated as quantitative texture descriptors for subsequent multivariate analysis.

A series of quantitative descriptors were extracted from the angle–energy curves, including peak intensity, peak position, peak width, curve area, spatial energy concentration, high-energy-band morphology, radial decay, entropy, contrast, correlation, and homogeneity. These variables characterize texture distribution, directional regularity, and frequency-domain energy concentration of CW surface images. The resulting energy spectrum features were combined with visible-color proxy features for subsequent multivariate analysis, feature screening, and regression modeling. Detailed procedures are summarized in Table S3.

### 2.3. Elemental Content Determination

Cu, Fe, Mn, and Zn were selected because they are essential trace elements with important nutritional and physiological functions. Fe is required for oxygen transport and energy metabolism, Cu contributes to iron utilization and the activity of antioxidant enzymes, Mn acts as a cofactor for several metabolic and antioxidant enzymes, and Zn participates in immune function, protein synthesis, and nucleic-acid metabolism. These elements were also selected because their concentrations may vary with geographical origin, soil conditions, and cultivation environment, making them representative indicators for evaluating the mineral quality of CW. The present study was therefore designed as a targeted assessment of four nutritionally relevant elements. ICP-MS was utilized to quantify the concentrations of four trace elements (Cu, Fe, Mn, and Zn) in CW samples [20,21]. The sample preparation involved microwave-assisted digestion, wherein 0.5 g of precisely weighed dried CW powder was placed into a polytetrafluoroethylene (PTFE) digestion vessel. Subsequently, 6 mL of analytical-grade nitric acid and 2 mL of analytical-grade hydrogen peroxide were added to the vessel. Following a 30 min standing period, the vessel was subjected to a microwave digestion system. The digestion protocol was implemented with the following temperature and time settings: 50 °C for 5 min, 100 °C for 5 min, and 180 °C for 20 min. All were conducted at a power level of 800 W. Upon completion of the digestion process, the digestion vessel was allowed to cool to ambient temperature. The resulting digested solution was subsequently transferred into a 50 mL volumetric flask, diluted to the calibration mark with ultrapure water, thoroughly mixed, and reserved for subsequent analysis. Concurrently, blank control groups and standard reference materials were established to facilitate quality control, thereby ensuring the accuracy and reliability of the detection results. The parameters for ICP-MS detection were as follows: the generator power was set at 1300 W; the atomizer flow rate was maintained at 0.95 L/min; the plasma torch cooling gas flow rate was 17.0 L/min; the auxiliary gas flow rate was 1.20 L/min; the detector analog stage voltage was set to −2350 V; and the ion lens voltage was adjusted to 6.00 V. The detection was conducted in peak-skipping mode. External calibration curves were constructed separately for the four elements. The coefficients of determination were R^2^ = 0.999836 for Cu, R^2^ = 0.999756 for Fe, R^2^ = 0.999875 for Mn, and R^2^ = 0.999667 for Zn, indicating satisfactory linearity within the respective working ranges. For each element, three replicate measurements were collected, and their mean was used as the final concentration for each batch.

### 2.4. Algorithm Principles of the Dung Beetle Optimizer-Radial Basis Function Regression Model

The Dung Beetle Optimizer-radial basis function (DBO-RBF) regression model was used to predict mineral element contents measured by inductively coupled plasma mass spectrometry (ICP-MS) from image-derived optical and texture features. The radial basis function (RBF) neural network provided the nonlinear mapping structure, while the Dung Beetle Optimizer (DBO) was used to optimize hidden-layer centers, kernel widths, and output-layer weights. This strategy was suitable for the present small-sample dataset because the RBF network can capture nonlinear relationships with a relatively compact structure, while DBO reduces the dependence on empirical parameter initialization.

Before model construction, each input variable was standardized using the mean and standard deviation of the training set:

$$x_{ik}^{\prime }=\frac{x_{ik}-\mu_{k}}{\sigma_{k}}$$

where x_{ik} is the original value of the k-th feature in the i-th sample, μ_k and σ_k are the mean and standard deviation calculated from the training samples, and x’_{ik} is the standardized value. The same training-set parameters were then applied to the test samples to avoid information leakage.

The RBF network contained an input layer, a hidden layer, and a linear output layer. For an input vector x_i, the activation of the j-th hidden node was calculated using a Gaussian radial basis function:

$$\varphi_{j\left(x_{i}\right)}=\exp\left(-\frac{∥x_{i}-c_{j}{∥}^{2}}{2\sigma_{j}^{2}}\right)$$

where c_j is the center vector of the j-th hidden node, σ_j is the kernel width, and ||x_i − c_j|| represents the Euclidean distance between the input sample and the hidden-node center. The predicted mineral element content was obtained by linearly combining the hidden-layer activations:

$${ŷ}_{i}=\sum_{j=1}^{m}w_{j}\varphi_{j\left(x_{i}\right)}+b$$

where m is the number of hidden nodes, w_j is the output weight of the j-th hidden node, b is the bias term, and ŷ_i is the predicted value.

In the DBO-RBF workflow, each individual in the DBO population encoded a candidate parameter vector:

$$\Theta =\left\{c_{1},c_{2},\ldots ,c_{m},\sigma_{1},\sigma_{2},\ldots ,\sigma_{m},w_{1},w_{2},\ldots ,w_{m},b\right\}$$

The optimization objective was to minimize the prediction error of the RBF model on the training samples. The root mean square error (RMSE) was used as the fitness function:

$$Fitness\left(\Theta \right)=RMSE_{train}=\sqrt{\frac{1}{n}\sum_{i=1}^{n}{\left(y_{i}-{ŷ}_{i}\right)}^{2}}$$

where n is the number of training samples, y_i is the measured mineral content, and ŷ_i is the predicted value. DBO iteratively updated the candidate parameter vectors through population-based global search and local refinement. The optimal parameter set was defined as

$$\Theta^{*}=\arg\underset{\Theta }{\min}Fitness\left(\Theta \right)$$

The optimized parameter set Θ* was then used to construct the final RBF regression model for predicting Cu, Fe, Mn, and Zn.

Model performance was evaluated using the coefficient of determination (R^2^), RMSE, mean absolute error (MAE), and ratio of performance to deviation (RPD):

$$R^{2}=1-\frac{\sum_{i=1}^{n}{\left(y_{i}-{ŷ}_{i}\right)}^{2}}{\sum_{i=1}^{n}{\left(y_{i}-\overset{-}{y}\right)}^{2}}$$

$$RMSE=\sqrt{\frac{1}{n}\sum_{i=1}^{n}{\left(y_{i}-{ŷ}_{i}\right)}^{2}}$$

$$MAE=\frac{1}{n}\sum_{i=1}^{n}\left|y_{i}-{ŷ}_{i}\right|$$

$$RPD=\frac{SD_{y}}{RMSE}$$

where ȳ is the mean measured value and SD_y is the standard deviation of the measured values. In this study, the 570–593 nm color-proxy band and the 0–40°/63–140° texture-angle features were used as input variables, and Cu, Fe, Mn, and Zn concentrations were modeled separately (see Table S4 for detailed algorithm steps).

### 2.5. Comparative Regression Algorithms

To further evaluate the predictive performance of DBO-RBF, nine commonly used regression algorithms were included as comparison models: K-nearest neighbors (KNN), Extremely Randomized Trees (Extra Trees), Random Forest (RF), support vector regression with a radial basis function kernel (SVR-RBF), eXtreme Gradient Boosting (XGBoost), standard radial basis function regression (standard RBF), Ridge regression, Multilayer Perceptron (MLP) regression, and Partial Least Squares Regression (PLSR). All models used the same input feature set and the same Cu, Fe, Mn, and Zn reference values.

KNN regression predicted mineral content according to the response values of neighboring samples in the feature space. Extra Trees and Random Forest were used as tree-based ensemble models. Random Forest constructed multiple decision trees from bootstrap samples, whereas Extra Trees introduced additional randomness during feature splitting. XGBoost was used as a Gradient Boosting model that sequentially reduced residual errors through additive tree construction.

SVR-RBF was included to evaluate kernel-based nonlinear regression. Standard RBF regression was used as the unoptimized RBF reference model, allowing the effect of DBO-based parameter optimization to be assessed. Ridge regression served as a regularized linear baseline. PLSR was included because it is widely used in spectral and chemometric modeling when predictor variables are correlated. MLP regression was used as a neural-network baseline with nonlinear fitting capacity.

All comparison models were evaluated using the same training–test partition and internal cross-validation strategy. The predictive performance of each model was reported using R^2^, RMSE, MAE, and RPD. These metrics were calculated separately for each mineral element to compare model stability across Cu, Fe, Mn, and Zn. The detailed settings and comparative results of the regression algorithms are summarized in Table S5.

### 2.6. Data Analysis

Experimental data are presented as mean ± standard deviation. Before conducting parametric comparisons, the normality of the data within each geographical group was assessed using the Shapiro–Wilk test, and the homogeneity of variances was evaluated using Levene’s test. Variables satisfying both assumptions were analyzed by one-way analysis of variance, followed by Duncan’s multiple-range test for pairwise comparisons. Statistical significance was defined as *p* < 0.05. For variables that did not meet the assumptions after appropriate transformation, the Kruskal–Wallis test followed by adjusted pairwise comparisons was used. Technical replicate measurements were averaged within each batch before multivariate analysis, yielding one observation for each of the 120 batches. PCA was performed using the complete individual batch-level data matrix rather than a table containing four regional means. All variables were autoscaled to a mean of zero and a standard deviation of one before PCA to prevent variables with larger numerical ranges from dominating the principal components. To facilitate visualization of within-group dispersion and intergroup overlap in the PCA and PLS-DA score plots, 95% confidence ellipses were calculated separately for each geographical origin from the individual sample scores and the corresponding within-group covariance matrix. These ellipses were used for graphical interpretation only and were not treated as an independent statistical test of group separation. PLS-DA was performed using the same sample-level matrix, with the geographical origins used as class labels. Variables with VIP values greater than 1 were selected as candidate features. The selected 570–593 nm color-proxy band and 0–40°/63–140° texture-angle features were then used to predict Cu, Fe, Mn, and Zn concentrations. The 120 samples were first divided into training and test sets at a ratio of 7:3. All preprocessing and scaling parameters were estimated exclusively from the training set and subsequently applied to the test set to prevent information leakage. Internal cross-validation was conducted within the training set to assess model stability. DBO-RBF was compared with KNN, Extra Trees, RF, SVR-RBF, XGBoost, standard RBF, Ridge regression, MLP regression, and PLSR. R^2^, RMSE, MAE, and RPD were used to evaluate regression performance. DBO-RBF modeling and comparative regression analysis were conducted using MATLAB R2023a and Python 3.10.12.

## 3. Results and Discussion

### 3.1. Image-Derived Visible-Color Proxy Characteristics and Regional Differentiation of CW

Standardized RGB images were used to extract image-derived visible-color proxy (VCP) features from CW samples. The analytical workflow is shown in Figure S1A. After image preprocessing and background removal, the effective fruit region was segmented and the mean RGB information was obtained. The RGB values were then converted into CIE L*a*b* color variables to describe lightness and chromatic characteristics. Subsequently, Gaussian color-band functions were configured across the visible range of 380–780 nm, and the band responses were superimposed and normalized to reconstruct a continuous VCP curve. This process transformed the original image color information into structured visible-range feature variables for subsequent multivariate analysis.

**Figure 2:**
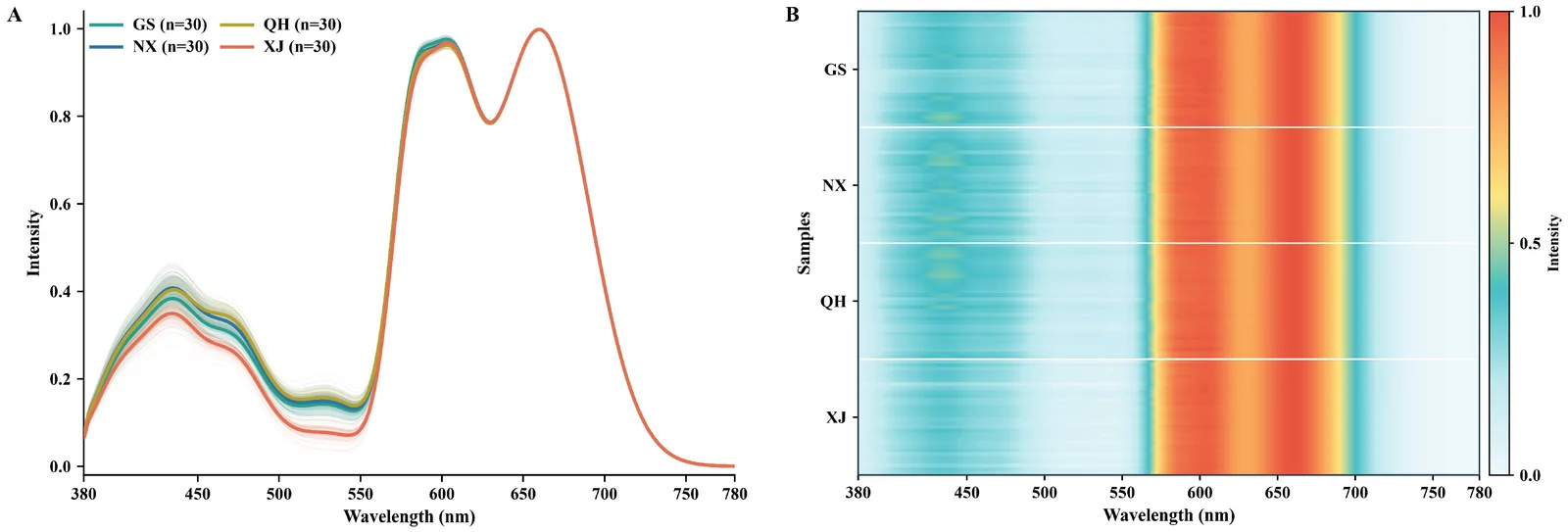
Image-derived visible-color proxy features of CW samples. Reconstructed visible-color proxy curves for CW samples from the four production areas (**A**); sample-level heatmap showing comparative visible-color proxy patterns among individual samples from the four production areas (**B**). QH, Qinghai; XJ, Xinjiang; NX, Ningxia; GS, Gansu.

As shown in Figure 2A, the reconstructed VCP curves of CW samples from Gansu (GS), Ningxia (NX), Qinghai (QH), and Xinjiang (XJ) showed a stable and highly consistent multi-peak profile. Three main response regions were observed, including the blue–violet region around 400–450 nm, the orange–yellow region around 580–630 nm and the orange–red region around 640–680 nm. These intervals were identified from the group-mean reconstructed visible-color proxy curves. Local maxima were located across the 380–780 nm range for each production area, and wavelength intervals containing reproducible maxima across all four origins were retained; the interval boundaries were defined by the adjacent local minima. Thus, the three regions represent common high-response intervals in the image-derived curves. These regions corresponded well to the visible appearance of mature CW fruits, which is mainly characterized by a reddish-orange surface color. Previous studies have shown that visible-color variation in plant materials is closely related to pigment accumulation and surface optical properties, including flavonoids, polyphenols, anthocyanins, and carotenoid-related compounds [29,30,31]. The strong response in the orange–yellow and orange–red regions indicates that RGB-derived VCP features effectively captured the dominant external color phenotype of CW samples.

Although the four origins shared a similar overall curve profile, clear local differences were observed in response intensity and curve morphology (Figure 2A). In the 400–450 nm region, GS, NX, and QH showed slightly higher responses than XJ, whereas XJ displayed a stronger response in the orange–red region, around 650–680 nm. GS, NX, and QH were more closely distributed in the main high-intensity region, while XJ showed a distinguishable orange–red response pattern. These differences indicate that the reconstructed VCP features retained origin-related optical phenotype information and provided useful variables for regional discrimination.

The sample-level heatmap further confirmed the structured distribution of VCP features across origins (Figure 2B). Most samples showed relatively low-to-moderate responses in the 380–550 nm region and strong responses in the 580–700 nm region. Meanwhile, the intensity bands and transition patterns differed among the GS, NX, QH, and XJ samples, showing that origin-related variation was consistently retained at the individual-sample level. The high-response interval around 570–700 nm was particularly prominent and provided an important color-response region for subsequent multivariate classification and feature-importance analysis.

Overall, Figure 2 demonstrates that RGB-derived VCP features provide a structured and non-destructive representation of CW external color phenotypes. The reconstructed curves and heatmap jointly reveal shared color characteristics and origin-related local differences among CW samples. These results support the use of VCP features for subsequent origin differentiation and quality-related modeling [1,2,24].

### 3.2. Frequency-Domain Texture Characteristics and Regional Variation in CW Images

Fourier transform analysis was used to characterize frequency-domain texture features of CW samples from different origins [27]. As shown in Figure S1B, standardized CW images were processed through image preprocessing, grayscale conversion, Fourier transform, two-dimensional spectrum mapping, angle–energy curve generation, and feature quantification. This workflow converted image texture information into structured frequency-domain descriptors, including angular energy distribution and spectrum-map features, thereby providing quantitative variables for origin-related texture comparison.

**Figure 3:**
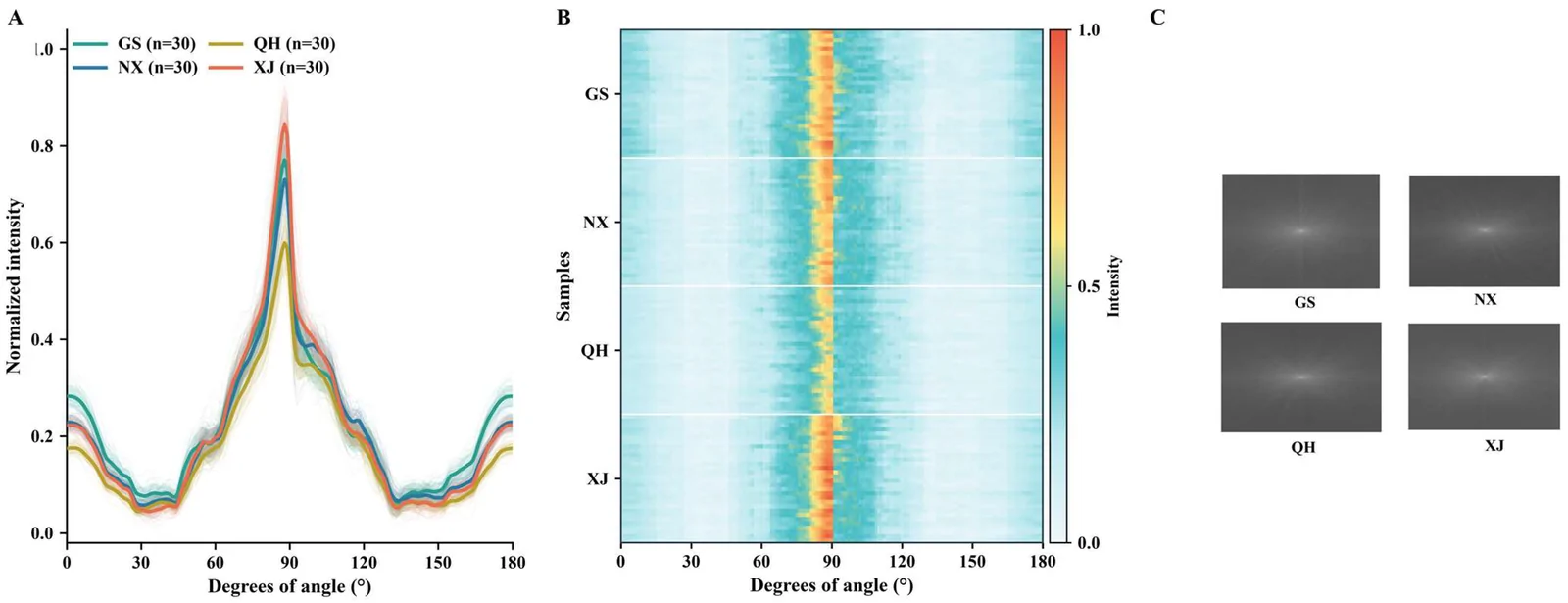
Frequency-domain texture characteristics of CW samples. Fourier-derived angle–energy curves for CW samples from the four production areas (**A**); sample-level heatmap of angular energy distributions (**B**); representative two-dimensional Fourier spectrum maps for samples from GS, NX, QH, and XJ (**C**). QH, Qinghai; XJ, Xinjiang; NX, Ningxia; GS, Gansu.

As shown in Figure 3A, the Fourier-derived angle–energy curves of CW samples from Gansu (GS), Ningxia (NX), Qinghai (QH), and Xinjiang (XJ) presented a consistent dominant peak near 90°, indicating that the main texture energy was concentrated around this angular direction. Meanwhile, the four origins showed distinguishable differences in peak height, curve width, and side-band distribution. XJ-CW exhibited the strongest main peak and a relatively sharp response near 90°, reflecting a more concentrated directional texture pattern. GS-CW also showed a strong angular energy response, with a slightly broader curve around the main peak. NX-CW displayed an intermediate response intensity, whereas QH-CW showed a lower and smoother peak. Overall, the main peak intensity followed the order XJ > GS > NX > QH.

The sample-level heatmap further confirmed the structured distribution of Fourier angle–energy features across origins (Figure 3B). A high-intensity vertical band was observed around 80–95° in most CW samples, consistent with the main peak shown in the mean angle–energy curves. At the same time, the intensity, width, and continuity of this dominant band varied among GS, NX, QH, and XJ. XJ samples showed a stronger and more concentrated high-energy band, GS samples presented a continuous and relatively broad energy response, NX samples showed moderate intensity, and QH samples displayed a smoother and weaker distribution. These heatmap patterns demonstrate that origin-related texture variation was consistently retained at the individual-sample level.

The representative two-dimensional Fourier spectrum maps provided additional visual evidence for origin-dependent texture differences (Figure 3C). The spectra of GS, NX, QH, and XJ all showed a central energy concentration with directional extension, while the brightness, spread, and angular distribution of the energy signals differed among origins. XJ showed a more pronounced central and directional energy pattern, GS displayed a relatively continuous energy distribution, NX exhibited moderate spectral intensity, and QH showed a softer texture energy profile. These differences were consistent with the curve and heatmap results.

Overall, Figure 3 demonstrates that Fourier-derived texture features effectively captured the frequency-domain characteristics of CW sample images. The angle–energy curves, sample-level heatmap, and representative two-dimensional spectra jointly revealed shared texture directionality and origin-related differences among CW samples. These results support the use of frequency-domain texture features for subsequent origin differentiation, feature selection, and mineral element prediction [27].

### 3.3. Mineral Element Content Characteristics and Regional Variation Patterns of CW from Different Origins

**Figure 4:**
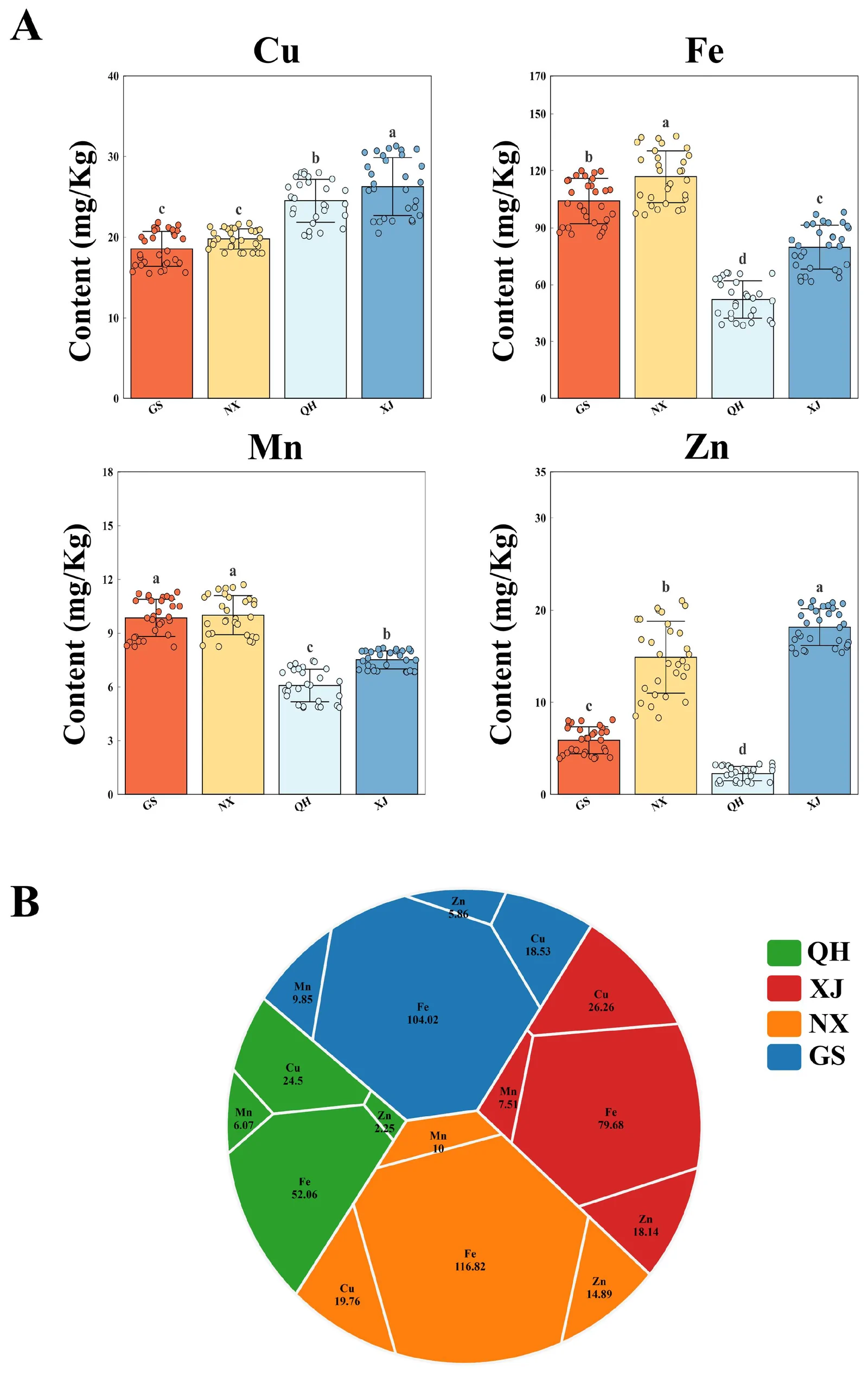
Mineral element distribution in CW from the four production areas. Contents of Cu, Fe, Mn, and Zn; different lowercase letters denote significant differences among production areas for the same element (*p* < 0.05) (**A**). Circular composition plot of the four mineral elements by production area; sector area is proportional to absolute element content (mg/kg) (**B**). QH, Qinghai; XJ, Xinjiang; NX, Ningxia; GS, Gansu.

Mineral elements serve as fundamental indicators for assessing the nutritional quality and authenticity of CW. Their concentrations and compositions are intricately influenced by the soil parent materials, climatic conditions, and cultivation practices prevalent in various regions. In this study, inductively coupled plasma mass spectrometry (ICP-MS) was employed to precisely quantify four key trace elements (Cu, Fe, Mn, Zn) in CW samples sourced from four principal production areas: GS, QH, XJ, and NX. Utilizing Duncan’s multiple range test (*p* < 0.05) in conjunction, the study systematically examined the impact of geographical origin on the composition and concentration of mineral elements in CW, with the findings presented in Figure 4. Figure 4A illustrates the regional variations in element concentrations and their statistical significance through a bar chart with letter annotations. The concentration of copper (Cu) is highest in XJ, followed by QH, NX, and GS, with significant differences observed between XJ/QH and NX/GS, but no significant differences within each subgroup. Iron (Fe), the most prevalent element in CW, exhibits the most pronounced regional variation, following the order NX > GS > XJ > QH, with significant differences among all four regions. Manganese (Mn) concentrations are ranked as NX > GS > XJ > QH, with no significant difference between NX and GS; however, both are significantly higher than XJ, which is significantly higher than QH. Zinc (Zn) concentrations decrease in the order of XJ > NX > GS > QH, with significant differences observed across all regions [32]. The circular composition plot in Figure 4B provides a detailed analysis of the elemental distribution characteristics of CW from various origins, based on absolute content. CW originating from GS exhibits the highest Fe content, with moderate levels of other elements, resulting in an overall balanced distribution. In contrast, CW from QH displays the lowest Fe content among the four regions, although Fe remains the predominant element. This is accompanied by low concentrations of Cu, Mn, and Zn, characterizing a “low total content but high Fe proportion” profile. CW from XJ is distinguished by significantly elevated Zn and Cu contents compared to other origins, along with moderate levels of Fe and Mn, indicating a nutritional profile characterized by “high Zn and high Cu.” Meanwhile, CW from NX is marked by the highest Fe and Mn contents among the four major origins, with moderate levels of Cu and Zn, establishing a dual-enrichment pattern of “high Fe and high Mn” with a balanced distribution of all elements. In summary, the four major production areas exhibit unique trace element profiles: NX-CW is enriched in Fe and Mn, XJ-CW has high Zn and Cu, GS-CW shows moderate and balanced elements, and QH-CW has a low total content but high Fe proportion [33,34]. These regional differences stem from the interplay of soil element backgrounds and climate on CW’s root absorption and transport mechanisms.

The XJ samples showed a distinct mineral profile characterized by the highest Cu and Zn concentrations. This enrichment is consistent with the combined influence of regional soil geochemistry and arid cultivation conditions on micronutrient availability. Soil parent material, pH, salinity, irrigation, and organic matter regulate the soluble and exchangeable fractions of Cu and Zn, while low rainfall and strong evaporation can alter salt distribution and element transport in the rhizosphere [15,17]. These processes provide a reasonable explanation for the XJ pattern, although their relative contributions could not be resolved because soil and irrigation-water properties were not measured.

### 3.4. Multivariate Clustering and Exploratory Marker Selection Based on Mineral, Color-Proxy, and Texture Features

**Figure 5:**
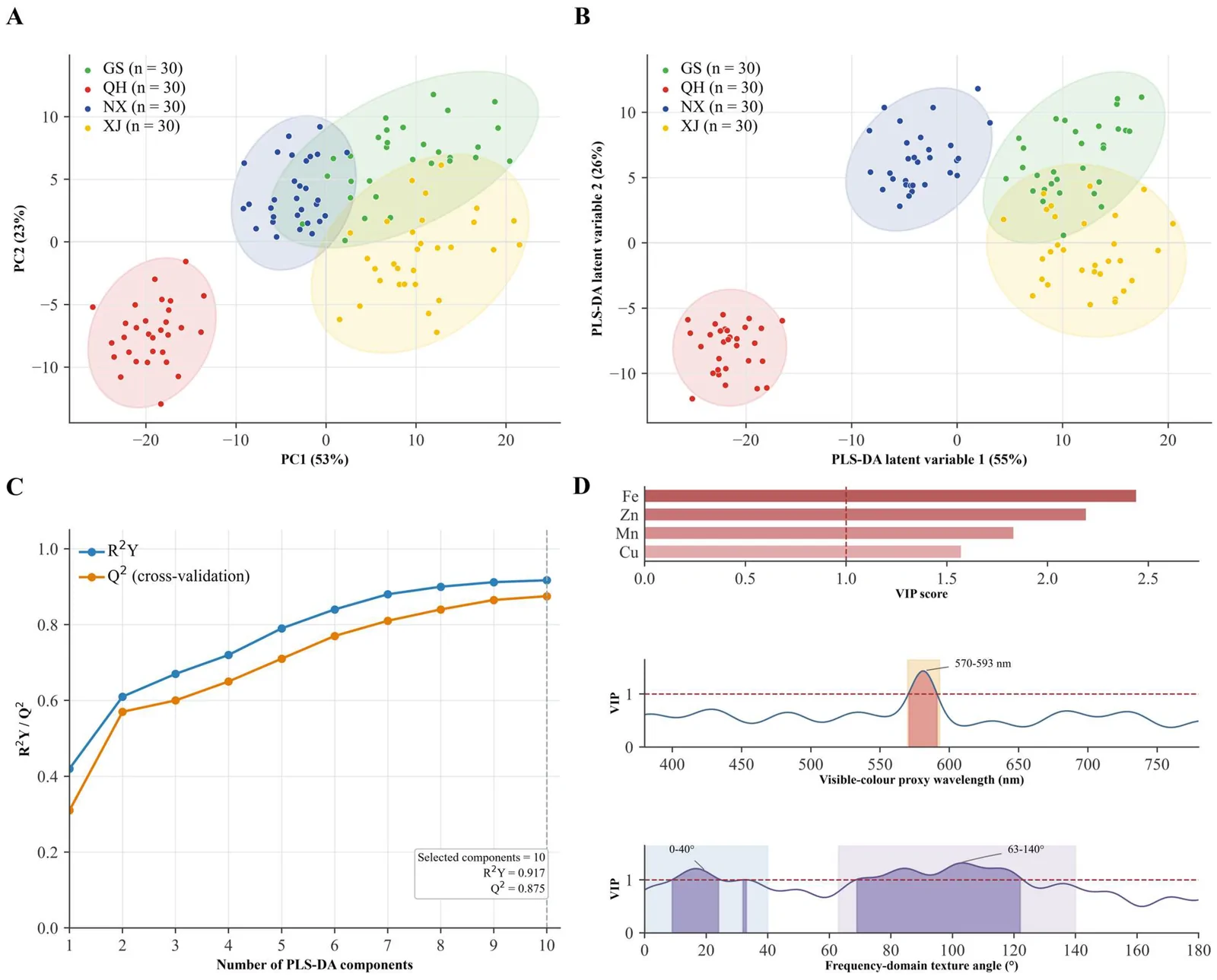
Multivariate differentiation of CW samples from the four production areas. PCA score plot (**A**) and PLS-DA score plot (**B**); shaded ellipses represent 95% confidence ellipses. R^2^Y and Q^2^ values with increasing numbers of PLS-DA components (**C**). VIP scores for mineral elements, the visible-color proxy band, and frequency-domain texture-angle features (**D**); dashed horizontal lines indicate the VIP = 1 threshold. QH, Qinghai; XJ, Xinjiang; NX, Ningxia; GS, Gansu.

Principal component analysis (PCA) and partial least squares discriminant analysis (PLS-DA) were used to evaluate the origin-related distribution of CW samples based on mineral elements, visible-color proxy features, and frequency-domain texture features. PCA was first applied as an unsupervised method to examine the natural clustering tendency of samples from Gansu (GS), Qinghai (QH), Ningxia (NX), and Xinjiang (XJ) [35]. As shown in Figure 5A, CW samples from different origins showed distinguishable distribution patterns in the PCA score plot. The PCA score plot indicated a tendency toward origin-related grouping rather than complete separation. QH samples were mainly distributed in the negative PC1 and PC2 region, whereas NX samples were concentrated in the upper-left region of the plot. GS and XJ samples were both located predominantly on the positive-PC1 side, with partially overlapping score distributions and 95% confidence ellipses. The first principal component explained 53% of the variance, and the second principal component explained 23%, with a cumulative contribution of 76%. Thus, the integrated feature matrix retained origin-related variation, but the PCA plot provides only descriptive evidence of distributional differences and does not, by itself, establish statistically significant separation or direct feature similarity among origins.

PLS-DA was further used to enhance supervised discrimination among origins [36]. Compared with PCA, the PLS-DA score plot showed clearer group separation and stronger within-group aggregation (Figure 5B). QH and NX were more distinctly separated, while the overlap between GS and XJ was reduced but not completely eliminated. The first two latent variables explained 55% and 26% of the variance, respectively, with a cumulative contribution of 81%. This result suggests that supervised modeling captured origin-related differences more effectively than unsupervised PCA, while also showing that some origins with similar feature profiles remained difficult to fully distinguish.

The performance of the PLS-DA model was further evaluated using R^2^ and Q^2^ (Figure 5C). R^2^ reflects the goodness of fit, whereas Q^2^ reflects predictive ability during validation. Both R^2^ and Q^2^ increased with the number of components, and Q^2^ remained positive, indicating that the model retained predictive ability rather than relying only on fitted variation. These results support the use of the PLS-DA model for exploratory origin discrimination and subsequent variable importance analysis.

Variable importance in projection (VIP) analysis was then used to identify candidate variables contributing to origin discrimination [37]. Variables with VIP > 1 were regarded as important contributors. As shown in Figure 5D, the mineral elements Fe, Zn, Mn, and Cu all exceeded the VIP threshold, indicating their important role in differentiating CW origins. Among the image-derived features, the 570–593 nm visible-color proxy band and the 0–40° and 63–140° texture-angle ranges also showed VIP values greater than 1. These results suggest that mineral profiles, visible-color proxy features, and frequency-domain texture features jointly contributed to the regional differentiation of CW. In particular, the 63–140° texture-angle range showed strong discriminative contribution, highlighting the value of frequency-domain texture descriptors in origin-related feature screening.

### 3.5. Internal Prediction of Key Mineral Element Contents Using the DBO-RBF Workflow

**Figure 6:**
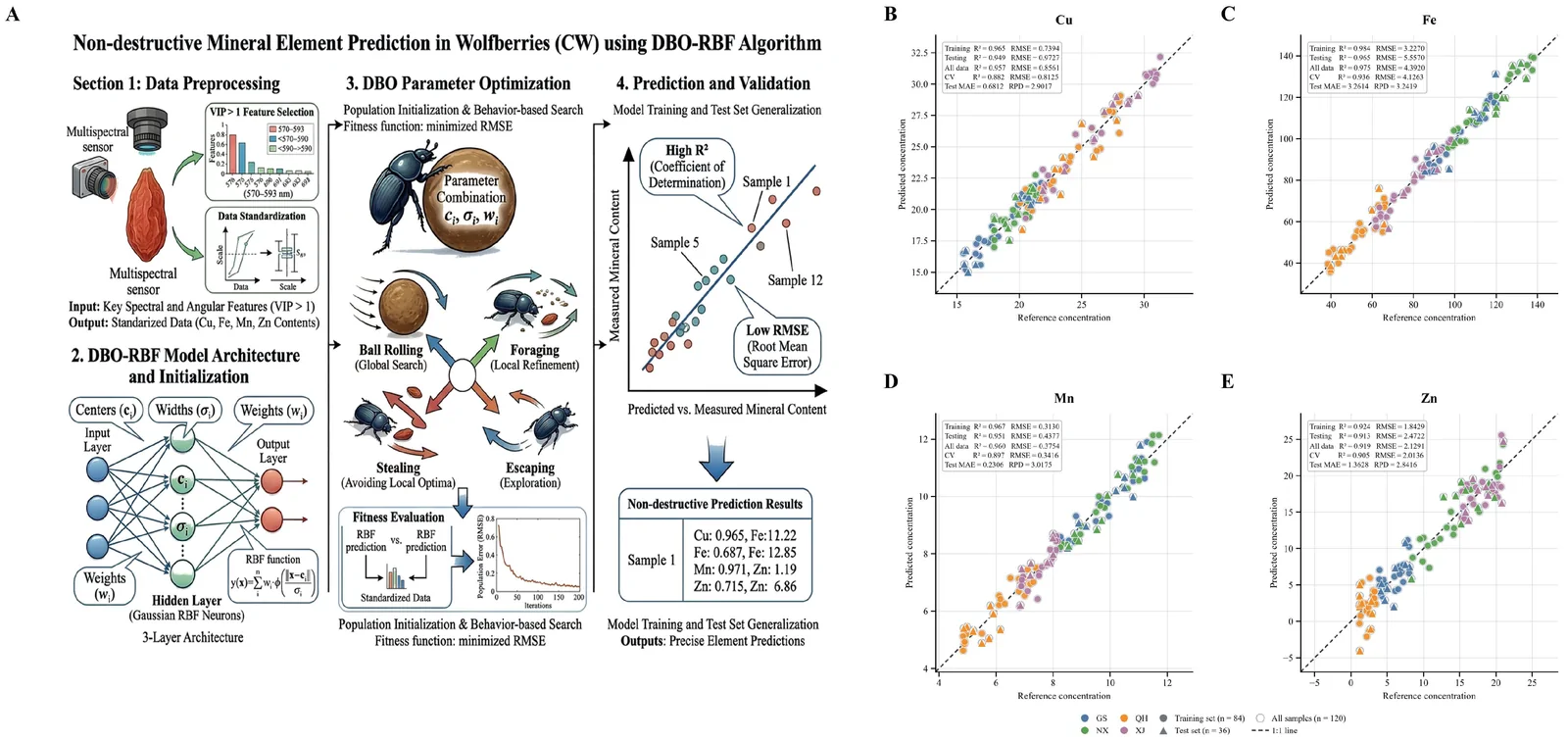
Internal prediction of Cu, Fe, Mn, and Zn contents in CW using the DBO-RBF workflow. Validation-aware workflow, including input-feature preparation, DBO-based parameter optimization, and model validation (**A**). Predicted versus reference concentrations for Cu (**B**), Fe (**C**), Mn (**D**), and Zn (**E**); colors indicate production area and symbols distinguish training, test, and full-dataset samples. Performance metrics were obtained from internal validation of the available dataset.

The DBO-RBF workflow was used to evaluate whether image-derived color-proxy and texture variables retained quantitative information related to ICP-MS-measured mineral element contents. Figure 6A summarizes the validation-aware analytical workflow, including training-set preprocessing, candidate feature selection, RBF network initialization, DBO-based parameter optimization, and independent prediction for training and test samples. The 570–593 nm color-proxy band and the 0–40° and 63–140° texture-angle features were used as input variables, while Fe, Zn, Mn and Cu concentrations served as output variables. This workflow enabled a direct assessment of the relationship between low-cost image-derived features and reference mineral measurements, providing a basis for evaluating their potential use in rapid preliminary mineral screening.

DBO-RBF showed strong internal prediction performance for all four mineral elements (Figure 6B–E). For Cu, the training, test, full-dataset, and cross-validation R^2^ values were 0.965, 0.949, 0.957, and 0.882, respectively, with corresponding RMSE values of 0.7394, 0.9727, 0.8561, and 0.8125 (Figure 6B). For Fe, the corresponding R^2^ values were 0.984, 0.965, 0.975, and 0.936, with RMSE values of 3.2270, 5.5570, 4.3920, and 4.1263 (Figure 6C). For Mn, R^2^ values were 0.967, 0.951, 0.960, and 0.897, with RMSE values of 0.3130, 0.4377, 0.3754, and 0.3416 (Figure 6D). For Zn, R^2^ values were 0.924, 0.913, 0.919, and 0.905, with RMSE values of 1.8429, 2.4722, 2.1576, and 2.0136 (Figure 6E). The test-set MAE values were 0.6812, 3.2614, 0.2306, and 1.3628 for Cu, Fe, Mn, and Zn, respectively, and the corresponding test RPD values were 2.9017, 3.2419, 3.0175, and 2.8416. These results indicate that the selected image-derived features preserved quantitative information related to mineral accumulation.

To assess whether this performance was specific to DBO-RBF, the model was compared with KNN, Extra Trees, Random Forest, SVR-RBF, XGBoost, standard RBF, Ridge, MLP, and PLSR under the same internal evaluation framework (Table 1). DBO-RBF achieved the highest test R^2^ for all four elements. Compared with the best non-DBO baseline, the test R^2^ increased from 0.715 to 0.949 for Cu, from 0.770 to 0.965 for Fe, from 0.748 to 0.951 for Mn, and from 0.798 to 0.913 for Zn. The same trend was observed for cross-validation R^2^, where DBO-RBF reached 0.882, 0.936, 0.897, and 0.905 for Cu, Fe, Mn, and Zn, respectively. In contrast, the best non-DBO cross-validation R^2^ values were 0.6278, 0.7510, 0.7305, and 0.8401. DBO-RBF also reduced test RMSE and MAE relative to the strongest baseline models, particularly for Cu, Fe, and Mn. The improvement for Zn was more moderate, indicating that Zn prediction remained the most challenging among the four elements.

These comparative results support the advantage of the DBO-RBF workflow for the present internal dataset (Table 1). Several baseline models, such as KNN, SVR-RBF, and XGBoost, showed very high training R^2^ but lower test or cross-validation performance, indicating overfitting under the limited sample size. DBO-RBF showed a better balance between fitting accuracy and internal validation performance, suggesting that DBO optimization improved the stability of the RBF model. Similar nonlinear regression and feature-fusion strategies have been reported as useful tools for agricultural product quality prediction and authenticity analysis [38,39,40]. Therefore, DBO-RBF provides a promising low-cost image-based screening strategy for mineral element estimation, while ICP-MS remains the reference method for confirmatory quantification. Previous Vis-NIR and hyperspectral studies of wolfberry have mainly used high-dimensional reflectance spectra for origin discrimination [1,2,22,24], whereas the present study used compact RGB-derived features for mineral prediction. Although these tasks are not directly comparable, the test-set R^2^ values of 0.913–0.965 and RPD values of 2.84–3.24 support the use of this lower-cost workflow for preliminary screening. ICP-MS remains necessary for confirmatory quantification.

The study has several limitations. Model performance was not evaluated using an independent external dataset, and the RGB features represent indirect optical phenotypes rather than mineral-specific chemical signals. The samples were collected from four regions in a single year, and all images were acquired under fixed conditions. Model transferability across years, production areas, imaging devices, and acquisition environments therefore requires validation using independent multi-year datasets and cross-device imaging experiments.

## 4. Conclusions

This study developed an image-derived workflow for the preliminary assessment of Cu, Fe, Mn, and Zn in CW. Standardized RGB imaging was used to reconstruct visible-color proxy features and derive frequency-domain texture descriptors, while ICP-MS provided the reference mineral concentrations. The integrated analysis showed that mineral profiles, the 570–593 nm color-proxy band, and the 0–40° and 63–140° texture-angle ranges jointly contributed to the differentiation of CW batches from Ningxia, Qinghai, Gansu, and Xinjiang. Thus, standardized external color and texture phenotypes retained screening-relevant information associated with regional mineral variation. Accordingly, H1 was supported by the observed regional differences in the four mineral concentrations, while H2 was supported within the present dataset by the associations between mineral variation and the selected image-derived features.

Using the VIP-selected image features, DBO-RBF achieved the strongest internal predictive performance among KNN, Extra Trees, Random Forest, SVR-RBF, XGBoost, standard RBF, Ridge, MLP, and PLSR. Its test-set R^2^ values reached 0.949, 0.965, 0.951, and 0.913 for Cu, Fe, Mn, and Zn, respectively. Compared with the best non-DBO model for each element, the corresponding R^2^ values increased from 0.715 to 0.949 for Cu, from 0.770 to 0.965 for Fe, from 0.748 to 0.951 for Mn, and from 0.798 to 0.913 for Zn. H3 was therefore supported because the VIP-selected features coupled with DBO-RBF consistently outperformed the comparative regression algorithms for all four elements. The workflow provides a low-cost, non-destructive approach for preliminary batch ranking and high-throughput CW screening. Nevertheless, RGB-derived features remain indirect optical correlates of mineral variation. Independent batches acquired under different imaging conditions should be evaluated to establish model transferability, while ICP-MS should remain the reference method for confirmatory mineral quantification.

## Figures and Tables

**Table 1:** Comparative prediction results of DBO-RBF and baseline regression algorithms for Cu, Fe, Mn, and Zn.

Element	Algorithm	Train R^2^	Test R^2^	Full R^2^	CV R^2^	Train RMSE	Test RMSE	Full RMSE	CV RMSE	Test MAE	CV MAE	Test RPD	CV RPD
Cu	DBO-RBF	0.9650	0.9490	0.9570	0.8820	0.7394	0.9727	0.8561	0.8125	0.6812	0.6035	2.9017	2.9154
Cu	KNN	0.9982	0.7150	0.8566	0.5310	0.0000	2.2152	1.1076	2.5121	1.7825	2.0942	1.8413	1.5603
Cu	Extra Trees	0.9671	0.6880	0.8276	0.5723	0.7215	2.3316	1.5266	2.5012	2.0532	2.1317	1.7401	1.5975
Cu	Random Forest	0.9016	0.6800	0.7908	0.5794	1.2427	2.3531	1.7979	2.4805	2.0456	2.1093	1.7245	1.6121
Cu	SVR-RBF	0.9981	0.6730	0.8356	0.5025	0.1842	2.3715	1.2779	2.6714	2.0547	2.1738	1.7105	1.4942
Cu	XGBoost	0.9885	0.6460	0.8173	0.6278	0.4342	2.4612	1.4477	2.3501	2.1407	1.9143	1.6441	1.6995
Cu	Standard RBF	0.4732	0.5750	0.5241	0.4530	2.7912	2.6732	2.7322	2.8745	2.2704	2.3112	1.5112	1.4443
Cu	Ridge	0.4201	0.4840	0.4521	0.3863	2.9269	2.8932	2.9101	3.0142	2.4853	2.3941	1.3842	1.3786
Cu	MLP	0.3015	0.2780	0.2898	0.2453	3.2013	3.3002	3.2508	3.3251	2.8521	2.5874	1.2072	1.2507
Cu	PLSR	0.7385	0.2560	0.4973	0.0512	1.9850	3.3382	2.6616	3.6907	2.8547	2.8675	1.1939	1.1222
Fe	DBO-RBF	0.9840	0.9650	0.9750	0.9360	3.2270	5.5570	4.3920	4.1263	3.2614	2.9157	3.2419	3.2761
Fe	Random Forest	0.9542	0.7700	0.8621	0.7510	5.7855	13.8223	9.8039	12.9832	10.9525	10.6243	2.0625	2.1117
Fe	Extra Trees	0.9823	0.7560	0.8692	0.7365	3.7802	14.2222	9.0012	13.3031	11.0420	10.7431	1.9984	2.0587
Fe	XGBoost	0.9948	0.7380	0.8664	0.7280	2.1929	14.7252	8.4591	13.4821	11.5329	10.7587	1.9307	2.0313
Fe	KNN	0.9990	0.7110	0.8550	0.7140	0.0000	15.4810	7.7405	13.6794	11.6063	11.2013	1.8417	1.9892
Fe	Standard RBF	0.6682	0.6750	0.6716	0.6328	15.1112	16.3501	15.7307	15.2013	13.2682	12.1610	1.7416	1.7863
Fe	SVR-RBF	0.9982	0.5380	0.7681	0.6162	1.2607	19.4082	10.3345	15.4922	14.2387	12.1701	1.4661	1.7533
Fe	Ridge	0.7552	0.4600	0.6076	0.4563	13.0846	20.8001	16.9424	18.3242	15.8093	14.2212	1.3710	1.4824
Fe	PLSR	0.8402	0.3390	0.5896	0.2575	10.6968	22.6701	16.6835	21.1701	17.8942	17.1261	1.2584	1.2818
Fe	MLP	0.8397	0.1550	0.4974	0.2778	10.7030	25.1121	17.9076	20.9042	18.3531	16.2101	1.1350	1.2982
Mn	DBO-RBF	0.9670	0.9510	0.9600	0.8970	0.3130	0.4377	0.3754	0.3416	0.2306	0.2041	3.0175	3.0428
Mn	Random Forest	0.9421	0.7480	0.8451	0.6875	0.4645	0.9121	0.6883	0.9812	0.7785	0.8074	1.9791	1.9311
Mn	Extra Trees	0.9825	0.7420	0.8623	0.6862	0.2765	0.9194	0.5980	0.9829	0.7676	0.8151	1.9633	1.9278
Mn	KNN	0.9985	0.7140	0.8563	0.7305	0.0000	0.9637	0.4819	0.9202	0.7942	0.7712	1.8629	2.0571
Mn	XGBoost	0.9924	0.6990	0.8457	0.6502	0.1842	0.9882	0.5862	1.0251	0.8672	0.8381	1.8167	1.8437
Mn	Standard RBF	0.6832	0.6410	0.6621	0.6154	1.0574	1.0721	1.0648	1.0690	0.9173	0.8815	1.6744	1.7713
Mn	SVR-RBF	0.9982	0.5870	0.7926	0.5325	0.0895	1.1465	0.6180	1.1619	0.9021	0.9413	1.5653	1.6244
Mn	Ridge	0.6682	0.5740	0.6211	0.5250	1.0785	1.1624	1.1205	1.1701	0.9243	0.9280	1.5441	1.6028
Mn	PLSR	0.8332	0.3540	0.5936	0.2528	0.7796	1.4112	1.0954	1.4604	1.1821	1.1957	1.2681	1.2805
Mn	MLP	0.5802	0.1190	0.3496	0.0648	1.2106	1.7331	1.4719	1.6271	1.3351	1.2701	1.0332	1.1542
Zn	DBO-RBF	0.9240	0.9130	0.9190	0.9050	1.8429	2.4722	2.1576	2.0136	1.3628	1.2194	2.8416	2.8672
Zn	Random Forest	0.9532	0.7980	0.8756	0.7942	1.5354	2.5741	2.0548	2.9542	2.0421	2.3101	2.5502	2.3201
Zn	Extra Trees	0.9882	0.7870	0.8876	0.8031	0.8542	2.6642	1.7592	2.8961	2.0571	2.2911	2.4641	2.3671
Zn	SVR-RBF	0.9983	0.7780	0.8882	0.8262	0.3211	2.7191	1.5201	2.7531	2.2041	2.1241	2.4131	2.4821
Zn	KNN	0.9988	0.7720	0.8854	0.8401	0.0000	2.7582	1.3791	2.6631	2.0491	1.9121	2.3791	2.5671
Zn	XGBoost	0.9972	0.7650	0.8811	0.7502	0.4521	2.8001	1.6261	3.1912	2.1061	2.3941	2.3431	2.1341
Zn	Standard RBF	0.7642	0.6580	0.7111	0.6942	3.3391	3.5842	3.4617	3.4991	2.9321	2.7631	1.8371	1.9641
Zn	MLP	0.9032	0.6430	0.7731	0.4802	2.2141	3.6671	2.9406	4.4721	3.0271	3.4911	1.7941	1.5341
Zn	Ridge	0.6802	0.6250	0.6526	0.5722	3.8541	3.7701	3.8121	4.0641	2.9931	3.3341	1.7471	1.6921
Zn	PLSR	0.8482	0.5870	0.7176	0.4442	2.7211	3.9621	3.3416	4.5791	3.1481	3.6971	1.6631	1.5091

Note: DBO-RBF, Dung Beetle Optimizer-radial basis function regression; KNN, K-nearest neighbors; SVR-RBF, support vector regression with radial basis function kernel; XGBoost, eXtreme Gradient Boosting; MLP, Multilayer Perceptron; PLSR, Partial Least Squares Regression.

## Data Availability

The data presented in this study are available on request from the corresponding authors (the data are not publicly available due to privacy).

## Supplementary Materials

The following supporting information can be downloaded at https://www.mdpi.com/article/10.3390/foods15162786/s1, Figure S1. Workflow for visible-color spectral proxy reconstruction and frequency-domain texture feature extraction from Chinese wolfberry images. Table S1. Geographic region information for Chinese wolfberry. Table S2. The specific steps of visible-color spectral proxy reconstruction and feature parameter extraction using the Python algorithm. Table S3. The specific steps of frequency-domain texture feature extraction using the Python algorithm. Table S4. The specific steps of the DBO-RBF regression workflow.Table S5. Detailed settings and implementation framework of the comparative regression algorithms.

## Abbreviations

CW, Chinese wolfberry; DBO, Dung Beetle Optimizer; DBO-RBF, Dung Beetle Optimizer-radial basis function; FFT, fast Fourier transform; FT, Fourier transform; ICP-MS, inductively coupled plasma mass spectrometry; KNN, K-nearest neighbors; LIBS, laser-induced breakdown spectroscopy; LSD, least significant difference; MAE, mean absolute error; MLP, Multilayer Perceptron; PCA, principal component analysis; PLS-DA, partial least squares discriminant analysis; PLSR, Partial Least Squares Regression; RBF, radial basis function; RF, Random Forest; RGB, red–green–blue; RMSE, root mean square error; RPD, ratio of performance to deviation; SD, standard deviation; SVR-RBF, support vector regression with a radial basis function kernel; VIP, variable importance in projection; XGBoost, eXtreme Gradient Boosting; XRF, X-ray fluorescence.
