# Supplementary material for: Visible-Spectrum Proxy and Texture Features Coupled with Optimized Regression for Predicting Key Minerals in Chinese Wolfberry
Source: Foods. 2026 Aug 8;15(16):2786. doi: 10.3390/foods15162786 (PMC13511821; doi:10.3390/foods15162786)
Supplement: Supplementary file 1 [file foods-15-02786-s001.zip › foods-4441304-supplementary.pdf]

Supplementary materials

Table S1 Geographic region information for Chinese wolfberry.

NO.	Name	Province	Longitude (˚E)	Latitude (˚N)	Altitude(m)	Batch number
1	CW	Xinjiang	73.40-96.50	34.22-48.10	461	3723912099
2	CW	Xinjiang	73.40-96.50	34.22-48.10	461	1979423896
3	CW	Xinjiang	73.40-96.50	34.22-48.10	461	3653044229
4	CW	Xinjiang	73.40-96.50	34.22-48.10	461	9305053602
5	CW	Xinjiang	73.40-96.50	34.22-48.10	461	6672347196
6	CW	Xinjiang	73.40-96.50	34.22-48.10	461	8546863882
7	CW	Xinjiang	73.40-96.50	34.22-48.10	461	6220064214
8	CW	Xinjiang	73.40-96.50	34.22-48.10	461	1946858162
9	CW	Xinjiang	73.40-96.50	34.22-48.10	461	8778632148
10	CW	Xinjiang	73.40-96.50	34.22-48.10	461	8809449186
11	CW	Xinjiang	73.40-96.50	34.22-48.10	461	3389599819
12	CW	Xinjiang	73.40-96.50	34.22-48.10	461	4377311225
13	CW	Xinjiang	73.40-96.50	34.22-48.10	461	5158455329
14	CW	Xinjiang	73.40-96.50	34.22-48.10	461	8064360206
15	CW	Xinjiang	73.40-96.50	34.22-48.10	461	9787356733
16	CW	Xinjiang	73.40-96.50	34.22-48.10	461	8608299721
17	CW	Xinjiang	73.40-96.50	34.22-48.10	461	6403164444
18	CW	Xinjiang	73.40-96.50	34.22-48.10	461	3601589450
19	CW	Xinjiang	73.40-96.50	34.22-48.10	461	8664078664
20	CW	Xinjiang	73.40-96.50	34.22-48.10	461	6684630013
21	CW	Xinjiang	73.40-96.50	34.22-48.10	461	6144612278
22	CW	Xinjiang	73.40-96.50	34.22-48.10	461	2948572707
23	CW	Xinjiang	73.40-96.50	34.22-48.10	461	6427636799
24	CW	Xinjiang	73.40-96.50	34.22-48.10	461	8035145828
25	CW	Xinjiang	73.40-96.50	34.22-48.10	461	5465458087
26	CW	Xinjiang	73.40-96.50	34.22-48.10	461	3641920390
27	CW	Xinjiang	73.40-96.50	34.22-48.10	461	9835658981
28	CW	Xinjiang	73.40-96.50	34.22-48.10	461	7018982068
29	CW	Xinjiang	73.40-96.50	34.22-48.10	461	8574481389
30	CW	Xinjiang	73.40-96.50	34.22-48.10	461	4586352944
31	CW	Qinghai	89.35-103.04	31.36-39.19	2676	1024701155
32	CW	Qinghai	89.35-103.04	31.36-39.19	2676	5250671586
33	CW	Qinghai	89.35-103.04	31.36-39.19	2676	9646614691
34	CW	Qinghai	89.35-103.04	31.36-39.19	2676	5215008285
35	CW	Qinghai	89.35-103.04	31.36-39.19	2676	4866507383
36	CW	Qinghai	89.35-103.04	31.36-39.19	2676	3338590620
37	CW	Qinghai	89.35-103.04	31.36-39.19	2676	8619557072
38	CW	Qinghai	89.35-103.04	31.36-39.19	2676	6895718991
39	CW	Qinghai	89.35-103.04	31.36-39.19	2676	3028954513
40	CW	Qinghai	89.35-103.04	31.36-39.19	2676	9258284258

41	CW	Qinghai	89.35-103.04	31.36-39.19	2676	7228367595
42	CW	Qinghai	89.35-103.04	31.36-39.19	2676	7556243230
43	CW	Qinghai	89.35-103.04	31.36-39.19	2676	2003882329
44	CW	Qinghai	89.35-103.04	31.36-39.19	2676	1532452123
45	CW	Qinghai	89.35-103.04	31.36-39.19	2676	6009409851
46	CW	Qinghai	89.35-103.04	31.36-39.19	2676	4926914576
47	CW	Qinghai	89.35-103.04	31.36-39.19	2676	4112798840
48	CW	Qinghai	89.35-103.04	31.36-39.19	2676	5264485856
49	CW	Qinghai	89.35-103.04	31.36-39.19	2676	7567532410
50	CW	Qinghai	89.35-103.04	31.36-39.19	2676	4725029035
51	CW	Qinghai	89.35-103.04	31.36-39.19	2676	3510658459
52	CW	Qinghai	89.35-103.04	31.36-39.19	2676	8781850393
53	CW	Qinghai	89.35-103.04	31.36-39.19	2676	5190063970
54	CW	Qinghai	89.35-103.04	31.36-39.19	2676	3418181308
55	CW	Qinghai	89.35-103.04	31.36-39.19	2676	3944983405
56	CW	Qinghai	89.35-103.04	31.36-39.19	2676	2296316042
57	CW	Qinghai	89.35-103.04	31.36-39.19	2676	4948401942
58	CW	Qinghai	89.35-103.04	31.36-39.19	2676	8898773096
59	CW	Qinghai	89.35-103.04	31.36-39.19	2676	5045910550
60	CW	Qinghai	89.35-103.04	31.36-39.19	2676	7876736880
61	CW	Gansu	92.13-108.46	34.11-42.57	1735	4404526924
62	CW	Gansu	92.13-108.46	34.11-42.57	1735	1899907239
63	CW	Gansu	92.13-108.46	34.11-42.57	1735	9161529859
64	CW	Gansu	92.13-108.46	34.11-42.57	1735	1203177995
65	CW	Gansu	92.13-108.46	34.11-42.57	1735	5387051789
66	CW	Gansu	92.13-108.46	34.11-42.57	1735	4576970083
67	CW	Gansu	92.13-108.46	34.11-42.57	1735	5482769420
68	CW	Gansu	92.13-108.46	34.11-42.57	1735	3573004423
69	CW	Gansu	92.13-108.46	34.11-42.57	1735	4419447203
70	CW	Gansu	92.13-108.46	34.11-42.57	1735	6800228736
71	CW	Gansu	92.13-108.46	34.11-42.57	1735	4879440825
72	CW	Gansu	92.13-108.46	34.11-42.57	1735	4055489298
73	CW	Gansu	92.13-108.46	34.11-42.57	1735	9255022383
74	CW	Gansu	92.13-108.46	34.11-42.57	1735	2048480468
75	CW	Gansu	92.13-108.46	34.11-42.57	1735	8064075964
76	CW	Gansu	92.13-108.46	34.11-42.57	1735	3182423138
77	CW	Gansu	92.13-108.46	34.11-42.57	1735	4194823686
78	CW	Gansu	92.13-108.46	34.11-42.57	1735	9487481180
79	CW	Gansu	92.13-108.46	34.11-42.57	1735	8232711398
80	CW	Gansu	92.13-108.46	34.11-42.57	1735	7763340698
81	CW	Gansu	92.13-108.46	34.11-42.57	1735	4219172404
82	CW	Gansu	92.13-108.46	34.11-42.57	1735	6606935851
83	CW	Gansu	92.13-108.46	34.11-42.57	1735	5372656591
84	CW	Gansu	92.13-108.46	34.11-42.57	1735	2406075588

85	CW	Gansu	92.13-108.46	34.11-42.57	1735	2014913121
86	CW	Gansu	92.13-108.46	34.11-42.57	1735	8665364512
87	CW	Gansu	92.13-108.46	34.11-42.57	1735	7263403221
88	CW	Gansu	92.13-108.46	34.11-42.57	1735	1029039565
89	CW	Gansu	92.13-108.46	34.11-42.57	1735	9861694385
90	CW	Gansu	92.13-108.46	34.11-42.57	1735	9062727134
91	CW	Ningxia	104.17-107.39	35.14-39.23	1189	4072694889
92	CW	Ningxia	104.17-107.39	35.14-39.23	1189	8033959540
93	CW	Ningxia	104.17-107.39	35.14-39.23	1189	3933294797
94	CW	Ningxia	104.17-107.39	35.14-39.23	1189	1294410146
95	CW	Ningxia	104.17-107.39	35.14-39.23	1189	2743730650
96	CW	Ningxia	104.17-107.39	35.14-39.23	1189	3805979527
97	CW	Ningxia	104.17-107.39	35.14-39.23	1189	9668385571
98	CW	Ningxia	104.17-107.39	35.14-39.23	1189	3828110702
99	CW	Ningxia	104.17-107.39	35.14-39.23	1189	4005337042
100	CW	Ningxia	104.17-107.39	35.14-39.23	1189	1594947732
101	CW	Ningxia	104.17-107.39	35.14-39.23	1189	9429987502
102	CW	Ningxia	104.17-107.39	35.14-39.23	1189	5852577100
103	CW	Ningxia	104.17-107.39	35.14-39.23	1189	4394771792
104	CW	Ningxia	104.17-107.39	35.14-39.23	1189	1820541235
105	CW	Ningxia	104.17-107.39	35.14-39.23	1189	5965634298
106	CW	Ningxia	104.17-107.39	35.14-39.23	1189	9220081497
107	CW	Ningxia	104.17-107.39	35.14-39.23	1189	8253861627
108	CW	Ningxia	104.17-107.39	35.14-39.23	1189	2195055326
109	CW	Ningxia	104.17-107.39	35.14-39.23	1189	4736797366
110	CW	Ningxia	104.17-107.39	35.14-39.23	1189	1887453145
111	CW	Ningxia	104.17-107.39	35.14-39.23	1189	5509952542
112	CW	Ningxia	104.17-107.39	35.14-39.23	1189	2252662498
113	CW	Ningxia	104.17-107.39	35.14-39.23	1189	4127393110
114	CW	Ningxia	104.17-107.39	35.14-39.23	1189	3757940610
115	CW	Ningxia	104.17-107.39	35.14-39.23	1189	7182976276
116	CW	Ningxia	104.17-107.39	35.14-39.23	1189	5931870413
117	CW	Ningxia	104.17-107.39	35.14-39.23	1189	6162490786
118	CW	Ningxia	104.17-107.39	35.14-39.23	1189	5914188676
119	CW	Ningxia	104.17-107.39	35.14-39.23	1189	8848090218
120	CW	Ningxia	104.17-107.39	35.14-39.23	1189	5643595938

Table S2 The specific steps of visible-color spectral proxy reconstruction and feature parameter

extraction using the Python algorithm.

```
import cv2
import numpy as np
import os
import pandas as pd
from datetime import datetime
from PIL import Image  # Import PIL library for pixel extraction as per original logic


def rgb_to_xyz(r, g, b):
    """Convert RGB values to XYZ color coordinates"""
    r_normalized = r / 255.0
    g_normalized = g / 255.0
    b_normalized = b / 255.0

    def gamma_correction(value):
        if value > 0.04045:
            return ((value + 0.055) / 1.055) ** 2.4
        else:
            return value / 12.92

    r_corrected = gamma_correction(r_normalized)
    g_corrected = gamma_correction(g_normalized)
    b_corrected = gamma_correction(b_normalized)

    x = r_corrected * 0.4124 + g_corrected * 0.3576 + b_corrected * 0.1805
    y = r_corrected * 0.2126 + g_corrected * 0.7152 + b_corrected * 0.0722
    z = r_corrected * 0.0193 + g_corrected * 0.1192 + b_corrected * 0.9505

    x *= 100
    y *= 100
    z *= 100

    return x, y, z


def xyz_to_lab(x, y, z):
    """Convert XYZ color coordinates to CIE L*a*b*"""
    x_n = 95.047
    y_n = 100.0
    z_n = 108.883

    x_normalized = x / x_n
    y_normalized = y / y_n
```

```python
    z_normalized = z / z_n

    def f(t):
        if t > 0.008856:
            return t ** (1 / 3)
        else:
            return 7.787 * t + 16 / 116

    f_x = f(x_normalized)
    f_y = f(y_normalized)
    f_z = f(z_normalized)

    l = 116 * f_y - 16 if y_normalized > 0.008856 else 903.3 * y_normalized
    a = 500 * (f_x - f_y)
    b_val = 200 * (f_y - f_z)

    return l, a, b_val


def gaussian_response(wavelength, mu, sigma, amplitude):
    """Gaussian response function: calculate response value of single wavelength in specified band"""
    return amplitude * np.exp(-((wavelength - mu) ** 2) / (2 * sigma ** 2))


def calculate_spectral_responses(l, a, b_val):
    """Calculate average response values of 7 specified spectral bands based on L*a*b* values"""
    bands = [
        {"name": "Red", "range": (620, 750), "mu": 660, "sigma": 30, "base_amp": 0.45 + a / 100},
        {"name": "Orange", "range": (590, 620), "mu": 605, "sigma": 15, "base_amp": 0.3 + a / 200 + b_val / 200},
        {"name": "Yellow", "range": (570, 590), "mu": 580, "sigma": 12, "base_amp": 0.35 + b_val / 150},
        {"name": "Green", "range": (495, 570), "mu": 530, "sigma": 25, "base_amp": 0.35 - a / 100},
        {"name": "Blue", "range": (450, 495), "mu": 470, "sigma": 20, "base_amp": 0.35 - b_val / 100},
        {"name": "Indigo", "range": (420, 450), "mu": 435, "sigma": 15, "base_amp": 0.25 - b_val / 180 + (50 - l) / 200},
        {"name": "Violet", "range": (380, 420), "mu": 405, "sigma": 18, "base_amp": 0.2 - b_val / 200 + (60 - l) / 300}
    ]
```

```
    spectral_responses = {}
    total_max = 0
    temp_responses = []

    for band in bands:
        start, end = band["range"]
        wavelengths = np.arange(start, end + 1, 1)
        amplitude = max(0, band["base_amp"]) * (1 / 100)
        responses = [gaussian_response(λ, band["mu"], band["sigma"], amplitude) for λ in
wavelengths]
        band_sum = np.sum(responses)
        temp_responses.append(band_sum)
        total_max = max(total_max, band_sum)

    total_max = total_max if total_max > 0 else 1
    for i, band in enumerate(bands):
        normalized_response = temp_responses[i] / total_max
        spectral_responses[f"{band['name']}_Response"] = round(normalized_response, 6)

    return spectral_responses


def extract_sample_color(image_path):
    """
    Extract average RGBA of target area + generate target area mask according to PIL Logic
    Core modification: strict pixel comparison (RGBA must be completely different to be
target),
    integer accumulation and division, retain Alpha channel
    Returns: avg_rgba (average RGBA), target_mask (target mask), image_bgr (OpenCV format
image for annotation)
    """
    # 1. Read image with PIL and convert to RGBA format
    pil_img = Image.open(image_path).convert('RGBA')
    width, height = pil_img.size
    # Get base color of top-left corner (RGBA format, strict comparison)
    base_color = pil_img.getpixel((0, 0))  # (R, G, B, A)

    # 2. Traverse all pixels and filter target pixels (completely different from base color)
    target_pixels = []
    target_mask = np.zeros((height, width), dtype=bool)  # Mask: True=target pixel

    for x in range(width):
        for y in range(height):
```

```
            current_color = pil_img.getpixel((x, y))
            # Strict judgment: RGBA four channels must be completely different to be target
            if current_color != base_color:
                target_pixels.append(current_color)
                target_mask[y, x] = True  # Note: mask coordinates are (y,x), corresponding to
image (row, column)

    # 3. Handle extreme case: no target pixels (Whole image is consistent with base color)
    if len(target_pixels) == 0:
        print(f"[WARNING] All pixels of image [{os.path.basename(image_path)}] are consistent
with base color of top-left corner, "
              f"return base color as default value")
        avg_r, avg_g, avg_b, avg_a = base_color
        # Convert to OpenCV BGR format for subsequent annotation
        image_bgr = cv2.cvtColor(np.array(pil_img), cv2.COLOR_RGBA2BGR)
        return (avg_r, avg_g, avg_b, avg_a), target_mask, image_bgr

    # 4. Calculate average RGBA according to PIL logic: integer accumulation then division (no
floating-point average)
    total_r = total_g = total_b = total_a = 0
    for (r, g, b, a) in target_pixels:
        total_r += r
        total_g += g
        total_b += b
        total_a += a

    pixel_count = len(target_pixels)
    avg_r = total_r // pixel_count
    avg_g = total_g // pixel_count
    avg_b = total_b // pixel_count
    avg_a = total_a // pixel_count

    # 5. Convert to OpenCV BGR format (for subsequent target area annotation)
    image_bgr = cv2.cvtColor(np.array(pil_img), cv2.COLOR_RGBA2BGR)

    return (avg_r, avg_g, avg_b, avg_a), target_mask, image_bgr


def visualize_target_area(image_bgr, target_mask, save_dir, image_name):
    """Target area visualization: generate original image + red target box to help verify
target area accuracy"""
    if not os.path.exists(save_dir):
        os.makedirs(save_dir)
```

```
    img_with_box = image_bgr.copy()
    target_coords = np.argwhere(target_mask)  # Get all target pixel coordinates (row, column)

    if len(target_coords) > 0:
        y_min, x_min = target_coords.min(axis=0)
        y_max, x_max = target_coords.max(axis=0)
        cv2.rectangle(img_with_box, (x_min, y_min), (x_max, y_max), (0, 0, 255), 2)
        label = f"Target: ({x_min},{y_min}) → ({x_max},{y_max})"
        cv2.putText(img_with_box, label, (x_min, y_min - 10),
                    cv2.FONT_HERSHEY_SIMPLEX, 0.5, (0, 0, 255), 1)
    else:
        cv2.putText(img_with_box, "No Target Area",
                    (20, 50), cv2.FONT_HERSHEY_SIMPLEX, 0.8, (0, 0, 255), 2)

    box_save_path = os.path.join(save_dir, f"{image_name}_Target_Area_Annotation.jpg")
    cv2.imwrite(box_save_path, img_with_box)
    print(f"[OK] Target area annotation image saved: {os.path.basename(box_save_path)}")


def analyze_single_image(image_path, vis_save_dir):
    """Analyze single image, extract RGBA according to PIL logic, retain original color space
conversion and 7-band response calculation"""
    try:
        # Extract average RGBA of target area (according to PIL logic)
        avg_rgba, target_mask, image_bgr = extract_sample_color(image_path)
        avg_r, avg_g, avg_b, avg_a = avg_rgba  # Split RGBA components

        # Color space conversion (only use RGB components, consistent with original logic)
        x, y, z = rgb_to_xyz(avg_r, avg_g, avg_b)
        l, a, b_val = xyz_to_lab(x, y, z)

        # Calculate response values of 7 specified spectral bands
        spectral_responses = calculate_spectral_responses(l, a, b_val)

        # Generate target area visualization image
        image_name = os.path.splitext(os.path.basename(image_path))[0]
        visualize_target_area(image_bgr, target_mask, vis_save_dir, image_name)

        # Print analysis results (add Alpha channel information)
        print(f"\n=== Analysis Result of Sample [{image_name}] ===")
        print(f"Average RGBA of Target Area: R={avg_r:>3d}, G={avg_g:>3d}, B={avg_b:>3d},
A={avg_a:>3d}")
        print(f"CIE XYZ: X={x:.2f}, Y={y:.2f}, Z={z:.2f}")
        print(f"CIE L*a*b*: L*={l:.2f}, a*={a:.2f}, b*={b_val:.2f}")
```

```
        for band_name, response in spectral_responses.items():
            print(f"{band_name}: {response:.6f}")
        print("=" * 50)

        # Integrate data (add average Alpha value column)
        result_data = {
            "Image_Name": image_name,
            "Average_R": avg_r,
            "Average_G": avg_g,
            "Average_B": avg_b,
            "Average_A": avg_a,  # Add Alpha channel data
            "CIE_X": round(x, 6),
            "CIE_Y": round(y, 6),
            "CIE_Z": round(z, 6),
            "CIE_L*": round(l, 6),
            "CIE_a*": round(a, 6),
            "CIE_b*": round(b_val, 6)
        }
        result_data.update(spectral_responses)

        return result_data

    except Exception as e:
        print(f"\n[ERROR] Error analyzing image [{image_path}]: {str(e)}")
        return None


def export_to_excel(all_results, output_folder="Target_Area_Color_Spectral_Data"):
    """Export to Excel (add average Alpha value column)"""
    if not os.path.exists(output_folder):
        os.makedirs(output_folder)
        print(f"[OK] New output folder created: {output_folder}")

    if not all_results:
        print("[WARNING] No data to export")
        return None

    timestamp = datetime.now().strftime("%Y%m%d_%H%M%S")
    excel_filename = f"{output_folder}/Target_Area_Color_7Band_Response_Data_{timestamp}.xlsx"

    data_df = pd.DataFrame(all_results)
    # Adjust column order (add average A value column)
    column_order = [
        "Image_Name",
```

```
        "Average_R", "Average_G", "Average_B", "Average_A",  # Add Alpha column
        "CIE_X", "CIE_Y", "CIE_Z",
        "CIE_L*", "CIE_a*", "CIE_b*",
        "Red_Response", "Orange_Response", "Yellow_Response",
        "Green_Response", "Blue_Response", "Indigo_Response", "Violet_Response"
    ]
    data_df = data_df.reindex(columns=column_order, fill_value="")

    with pd.ExcelWriter(excel_filename, engine="openpyxl") as writer:
        data_df.to_excel(writer, sheet_name='Target_Area_Data', index=False)

    print(f"\n[OUTPUT] Data export completed! File path: {excel_filename}")
    print(f"[INFO] Contains: target area data (including Alpha channel) of {len(all_results)} images + 1 integrated worksheet")
    return excel_filename


def batch_analyze_folder(folder_path):
    """Batch analyze images in folder (logic consistent with original code, only rely on modified extraction function)"""
    if not os.path.exists(folder_path):
        raise ValueError(f"Folder does not exist: {folder_path} (Please confirm the path is correct)")

    vis_save_dir = os.path.join(folder_path, "Target_Area_Annotation")
    print(f"[INFO] Target area annotation images will be saved to: {vis_save_dir}")

    # Support common image formats readable by PIL
    image_extensions = ('.png', '.jpg', '.jpeg', '.PNG', '.JPG', '.bmp', '.tiff')
    image_paths = [
        os.path.join(folder_path, file_name)
        for file_name in os.listdir(folder_path)
        if file_name.lower().endswith(image_extensions)
    ]

    if len(image_paths) == 0:
        print(f"[WARNING] No supported image files found in folder [{folder_path}] (Formats: {image_extensions})")
        return []

    print(f"[OK] Found {len(image_paths)} images, start target area color and 7-band response analysis...")
    all_results = []
    for idx, img_path in enumerate(image_paths, 1):
```

```python
        print(f"\n--- Analyzing {idx}/{len(image_paths)} image ---")
        result = analyze_single_image(img_path, vis_save_dir)
        if result is not None:
            all_results.append(result)

    if all_results:
        export_to_excel(all_results)

    print(f"\n[SUMMARY] Batch analysis completed! Successfully processed
{len(all_results)}/{len(image_paths)} images")
    return all_results


# Main function entry
def main():
    # Please change the path here to the absolute path of your image folder (e.g.
"D:/image_folder")
    image_folder = "1"
    batch_analyze_folder(image_folder)


if __name__ == "__main__":
    main()
```

Table S3 The specific steps of frequency-domain texture feature extraction using the Python algorithm.

```
import os
import cv2
import numpy as np
import matplotlib.pyplot as plt

def halfcircle(r, x0, y0):
    theta = np.linspace(0, np.pi, 360)
    x = x0 + r * np.cos(theta)
    y = y0 + r * np.sin(theta)
    return x.astype(int), y.astype(int)

def intline(x1, x2, y1, y2):
    dx, dy = abs(x2-x1), abs(y2-y1)
    if dx == 0 and dy == 0:
        x, y = np.array([x1]), np.array([y1])
        return x, y
    if dx > dy:
        if x1 > x2:
            x1, x2 = x2, x1
            y1, y2 = y2, y1
        m = (y2-y1) / (x2-x1)
        x = np.arange(x1, x2+1, 1)  # [x1,x2]
        y = (y1 + m*(x-x1)).astype(int)
    else:
        if y1 > y2:
            x1, x2 = x2, x1
            y1, y2 = y2, y1
        m = (x2-x1) / (y2-y1)
        y = np.arange(y1, y2+1, 1)  # [y1,y2]
        x = (x1 + m*(y-y1)).astype(int)
    return x, y

def specxture(gray):
    height, width = gray.shape
    x0, y0 = int(height / 2), int(width / 2)
    rmax = min(height, width) // 2 - 1
    gray32 = np.float32(gray)
    dft = cv2.dft(gray32, flags=cv2.DFT_COMPLEX_OUTPUT)
    dftShift = np.fft.fftshift(dft)
    sAmp = cv2.magnitude(dftShift[:, :, 0], dftShift[:, :, 1])
    sAmpLog = np.log10(1 + np.abs(sAmp))
    sRad = np.zeros((rmax,))
```

```
    sRad[0] = sAmp[x0, y0]
    for r in range(1, rmax):
        xc, yc = halfcircle(r, x0, y0)
        sRad[r] = sum(sAmp[xc[i], yc[i]] for i in range(len(xc)))
    sRadLog = np.log10(1 + np.abs(sRad))
    xmax, ymax = halfcircle(rmax, x0, y0)
    sAng = np.zeros((len(xmax),))
    for a in range(len(xmax)):
        xr, yr = intline(x0, xmax[a], y0, ymax[a])
        sAng[a] = sum(sAmp[xr[i], yr[i]] for i in range(len(xr)))
    return sAmpLog, sRadLog, sAng


# Define the main function
def main():
    # Set the folder path for the images
    image_folder = "1"  # Folder containing the images
    image_files = os.listdir(image_folder)

    # Loop through each image and process it
    for img_file in image_files:
        img_path = os.path.join(image_folder, img_file)
        gray = cv2.imread(img_path, flags=0)
        if gray is None:
            print(f"Error loading image {img_file}.")
            continue  # Skip if the image file cannot be read

        # Process the image
        sAmpLog, sRadLog, sAng = specxture(gray)

        # Plot the results
        plt.figure(figsize=(9, 6))
        plt.subplot(241), plt.axis('off'), plt.title("Original Image"), plt.imshow(gray,
'gray')
        plt.subplot(242), plt.axis('off'), plt.title("Amp spectrum"), plt.imshow(sAmpLog,
'gray')
        plt.subplot(245), plt.plot(sRadLog), plt.title("S (radius)"), plt.xlim(0, 300),
plt.yticks([])
        plt.subplot(246), plt.plot(sAng), plt.title("S (theta)"), plt.xlim(0, 180),
plt.yticks([])
        plt.tight_layout()

        # Save the figure in the original folder with a new filename
        output_filename = os.path.splitext(img_file)[0] + '_output.png'
```

```
        output_path = os.path.join(image_folder, output_filename)
        plt.savefig(output_path)
        plt.close()  # Close the figure to free memory

        # Here you can add additional code to process or save sAmpLog, sRadLog, sAng
        # For example, you might want to save these arrays as text files or .npy files.


if __name__ == "__main__":
    main()
```

Table S4 The specific steps of the DBO-RBF regression workflow.

Cross-validation implementation note: The reported CV-R^2 was obtained by five-fold cross-validation within the 70% training set. For each fold, input/output normalization and DBO optimization were fitted using only the four training partitions, and the optimized model predicted the held-out partition. The pooled out-of-fold predictions were used to calculate CV-R^2 and the other CV metrics. The independent 30% test set was not used during cross-validation or optimization.

```
%% Clear environment variables
warning off             % Turn off warning messages
close all               % Close all open figure windows
clear                   % Clear variables
clc                     % Clear command window

%% Import data
res = xlsread('Dataset.xlsx'); % Import dataset (rename your Excel file to Dataset.xlsx)

%% Data analysis
train_ratio = 0.7;                          % Ratio of training set to total dataset
output_dim = 1;                             % Last column as output
num_samples = size(res, 1);                 % Number of samples
res = res(randperm(num_samples), :);        % Shuffle dataset (comment this line if not needed)
num_train_samples = round(train_ratio * num_samples); % Number of training samples
feature_dim = size(res, 2) - output_dim; % Input feature dimension

%% Split training and testing sets
P_train = res(1:num_train_samples, 1:feature_dim)'; % Training set inputs
T_train = res(1:num_train_samples, feature_dim + 1:end)'; % Training set outputs
M = size(P_train, 2);                       % Number of training samples

P_test = res(num_train_samples + 1:end, 1:feature_dim)'; % Testing set inputs
T_test = res(num_train_samples + 1:end, feature_dim + 1:end)'; % Testing set outputs
N = size(P_test, 2);                        % Number of testing samples

%% Data normalization
[p_train, ps_input] = mapminmax(P_train, 0, 1); % Normalize training inputs to [0,1]
p_test = mapminmax('apply', P_test, ps_input);   % Apply normalization to testing inputs

[t_train, ps_output] = mapminmax(T_train, 0, 1); % Normalize training outputs to [0,1]
t_test = mapminmax('apply', T_test, ps_output);   % Apply normalization to testing outputs

%% DBO parameter settings
pop_size = 800;                             % Population size
max_iter = 700;                             % Maximum iteration number
```

```
input_num = size(P_train, 1);                    % Number of input nodes
hidden_num = 5;                                    % Number of hidden layer nodes
dim = (input_num + 1) * hidden_num + hidden_num * output_dim; % Parameter dimension
lb = -5 * ones(1, dim);                          % Lower bound
ub = 5 * ones(1, dim);                            % Upper bound

%% Fitness function
fobj = @(x)objfun(x, p_train', t_train', input_num, hidden_num, output_dim);

%% Call DBO optimization
[Best_score, Best_pos, Curve] = DBO(pop_size, max_iter, lb, ub, dim, fobj);

%% Five-fold cross-validation within the training set
% DBO optimization and normalization are repeated independently inside each fold.
rng(42, 'twister');
num_folds = 5;
cv_part = cvpartition(M, 'KFold', num_folds);
T_cv = nan(size(T_train));
fold_R2 = nan(num_folds, 1);
fold_RMSE = nan(num_folds, 1);

for fold = 1:num_folds
    fit_idx = training(cv_part, fold);
    val_idx = test(cv_part, fold);

    P_fit = P_train(:, fit_idx);
    T_fit = T_train(:, fit_idx);
    P_val = P_train(:, val_idx);
    T_val = T_train(:, val_idx);

    [p_fit, ps_input_cv] = mapminmax(P_fit, 0, 1);
    p_val = mapminmax('apply', P_val, ps_input_cv);
    [t_fit, ps_output_cv] = mapminmax(T_fit, 0, 1);

    fobj_cv = @(x)objfun(x, p_fit', t_fit', input_num, hidden_num, output_dim);
    [~, Best_pos_cv] = DBO(pop_size, max_iter, lb, ub, dim, fobj_cv);
    val_sim_norm = Resultfun(Best_pos_cv, p_val', input_num, hidden_num, output_dim);

    % Resultfun returns samples by outputs; mapminmax expects outputs by samples.
    if size(val_sim_norm, 1) == sum(val_idx)
        val_sim_norm = val_sim_norm';
    end
    val_sim = mapminmax('reverse', val_sim_norm, ps_output_cv);
    T_cv(:, val_idx) = val_sim;
```

```
    fold_R2(fold) = 1 - sum((T_val(:) - val_sim(:)).^2) / ...
        sum((T_val(:) - mean(T_val(:))).^2);
    fold_RMSE(fold) = sqrt(mean((T_val(:) - val_sim(:)).^2));
end

% Pool all out-of-fold predictions to obtain the reported CV metrics.
cv_error = T_cv(:) - T_train(:);
R2_cv = 1 - sum((T_train(:) - T_cv(:)).^2) / ...
    sum((T_train(:) - mean(T_train(:))).^2);
rmse_cv = sqrt(mean(cv_error.^2));
mse_cv = mean(cv_error.^2);
MAE_cv = mean(abs(cv_error));
MBE_cv = mean(cv_error);
MAPE_cv = mean(abs(cv_error ./ T_train(:)));
RPD_cv = std(T_train(:)) / std(cv_error);

%%  Fitness curve - optimized version
figure('Position', [100, 100, 800, 400])
plot(1:length(Curve), Curve, 'LineWidth', 2.5, ...
    'Color', [0.2, 0.5, 0.8], 'LineStyle', '-', ...
    'Marker', 'o', 'MarkerSize', 6, 'MarkerFaceColor', [0.2, 0.5, 0.8], ...
    'MarkerIndices', 1:50:length(Curve));
title('DBO Optimization Fitness Curve', 'FontSize', 14, 'FontWeight', 'bold');
xlabel('Iteration Number', 'FontSize', 12);
ylabel('Fitness Value', 'FontSize', 12);
grid on
set(gca, 'FontSize', 11, 'LineWidth', 1.2);
% Set background color
ax = gca;
ax.Color = [0.95, 0.95, 0.97];

%% Prediction
train_sim = Resultfun(Best_pos, p_train', input_num, hidden_num, output_dim); % Training set prediction
test_sim = Resultfun(Best_pos, p_test', input_num, hidden_num, output_dim);     % Testing set prediction

%% Data denormalization
T_sim1 = mapminmax('reverse', train_sim, ps_output); % Denormalize training predictions
T_sim2 = mapminmax('reverse', test_sim, ps_output);     % Denormalize testing predictions

T_sim1 = T_sim1';
T_sim2 = T_sim2';
```

```
%% Test set regression plot - optimized version
figure('Position', [100, 100, 900, 400])
subplot(1,2,1)
scatter(T_test, T_sim2, 60, 'filled', ...
    'MarkerFaceColor', [0.3, 0.75, 0.6], ...
    'MarkerEdgeColor', [0.1, 0.5, 0.3], ...
    'LineWidth', 1.5);
hold on
% Add diagonal line
plot([min(T_test), max(T_test)], [min(T_test), max(T_test)], ...
    'Color', [0.8, 0.2, 0.2], 'LineWidth', 2.5, 'LineStyle', '--');
hold off
title('Test Set Regression Plot', 'FontSize', 13, 'FontWeight', 'bold');
xlabel('True Values', 'FontSize', 12);
ylabel('Predicted Values', 'FontSize', 12);
grid on

legend('Data Points', 'y = x Reference Line', 'Location', 'best');
set(gca, 'FontSize', 11, 'LineWidth', 1.2);

subplot(1,2,2)
histogram(T_test - T_sim2, 20, ...
    'FaceColor', [0.5, 0.7, 0.9], ...
    'EdgeColor', [0.2, 0.4, 0.6], ...
    'LineWidth', 1.5, ...
    'FaceAlpha', 0.8);
title('Test Set Error Histogram', 'FontSize', 13, 'FontWeight', 'bold');
xlabel('Prediction Error', 'FontSize', 12);
ylabel('Frequency', 'FontSize', 12);
grid on

set(gca, 'FontSize', 11, 'LineWidth', 1.2);

%%  Root Mean Square Error (RMSE)
rmse_train = sqrt(sum((T_sim1 - T_train).^2)./M);
rmse_test = sqrt(sum((T_test - T_sim2).^2)./N);

%% Coefficient of determination (R²)
R2_train = 1 - norm(T_train - T_sim1)^2 / norm(T_train - mean(T_train))^2;
R2_test = 1 - norm(T_test -  T_sim2)^2 / norm(T_test -  mean(T_test ))^2;

%% Mean Square Error (MSE)
mse_train = sum((T_sim1 - T_train).^2)./M;
```

```
mse_test = sum((T_sim2 - T_test).^2)./N;

%% Residual Prediction Deviation (RPD)
SE_train = std(T_sim1 - T_train);
RPD_train = std(T_train)/SE_train;

SE_test = std(T_sim2 - T_test);
RPD_test = std(T_test)/SE_test;

%% Mean Absolute Error (MAE)
MAE_train = mean(abs(T_train - T_sim1));
MAE_test = mean(abs(T_test - T_sim2));

%% Mean Bias Error (MBE)
MBE_train = sum(T_sim1 - T_train) ./ M;
MBE_test = sum(T_sim2 - T_test) ./ N;

%% Mean Absolute Percentage Error (MAPE)
MAPE_train = mean(abs((T_train - T_sim1)./T_train));
MAPE_test = mean(abs((T_test - T_sim2)./T_test));

%%  Training set plot - optimized version
figure('Position', [100, 100, 1000, 400])
subplot(1,2,1)
plot(1:M, T_train, 'r-', 'LineWidth', 2.5, 'Marker', 'o', 'MarkerSize', 6, 'MarkerFaceColor',
'r');
hold on
plot(1:M, T_sim1, 'b--', 'LineWidth', 2, 'Marker', 's', 'MarkerSize', 6, 'MarkerFaceColor',
'b');
hold off
legend('True Values', 'DBO-RBF Predicted Values', 'Location', 'best', 'FontSize', 11);
xlabel('Sample Index', 'FontSize', 12);
ylabel('Prediction Results', 'FontSize', 12);
title('Training Set Prediction Comparison', 'FontSize', 13, 'FontWeight', 'bold');
grid on

set(gca, 'FontSize', 11, 'LineWidth', 1.2);

%% Test set plot - optimized version
subplot(1,2,2)
plot(1:N, T_test, 'r-', 'LineWidth', 2.5, 'Marker', 'o', 'MarkerSize', 6, 'MarkerFaceColor',
'r');
hold on
```

```
plot(1:N, T_sim2, 'b--', 'LineWidth', 2, 'Marker', 's', 'MarkerSize', 6, 'MarkerFaceColor',
'b');
hold off
legend('True Values', 'DBO-RBF Predicted Values', 'Location', 'best', 'FontSize', 11);
xlabel('Sample Index', 'FontSize', 12);
ylabel('Prediction Results', 'FontSize', 12);
title('Test Set Prediction Comparison', 'FontSize', 13, 'FontWeight', 'bold');
grid on

set(gca, 'FontSize', 11, 'LineWidth', 1.2);

%% Test set error plot - optimized version
figure('Position', [100, 100, 800, 400])
subplot(1,2,1)
error_test = T_test - T_sim2;
plot(1:N, error_test, 'b-*', 'LineWidth', 1.5, 'MarkerSize', 8, ...
    'MarkerFaceColor', [0.3, 0.6, 0.9]);
xlabel('Test Set Sample Index', 'FontSize', 12);
ylabel('Prediction Error', 'FontSize', 12);
title('Test Set Prediction Error', 'FontSize', 13, 'FontWeight', 'bold');
grid on

set(gca, 'FontSize', 11, 'LineWidth', 1.2);
% Add zero reference line
hold on
plot([1, N], [0, 0], 'r--', 'LineWidth', 1.5);
hold off
legend('DBO-RBF Prediction Error', 'Zero Reference Line', 'Location', 'best');

%% Relative error plot - optimized version
subplot(1,2,2)
relative_error = (T_sim2 - T_test) ./ T_test * 100; % Convert to percentage
bar(1:N, relative_error, ...
    'FaceColor', [0.8, 0.4, 0.6], ...
    'EdgeColor', [0.5, 0.2, 0.4], ...
    'LineWidth', 1, ...
    'FaceAlpha', 0.7);
hold on
% Add average relative error line
mean_rel_error = mean(relative_error);
plot([1, N], [mean_rel_error, mean_rel_error], ...
    'k--', 'LineWidth', 2, 'Color', [0.2, 0.2, 0.2]);
hold off
legend('Relative Error (%)', sprintf('Average: %.2f%%', mean_rel_error), 'Location', 'best');
```

```
title('Test Set Relative Error', 'FontSize', 13, 'FontWeight', 'bold');
ylabel('Relative Error (%)', 'FontSize', 12);
xlabel('Sample Index', 'FontSize', 12);
xlim([1, N]);
grid on

set(gca, 'FontSize', 11, 'LineWidth', 1.2);

%% Linear fitting plot - optimized version
figure('Position', [100, 100, 1200, 400])

% Training set fitting plot
subplot(1,3,1)
scatter(T_train, T_sim1, 50, 'filled', ...
    'MarkerFaceColor', [0.9, 0.4, 0.4], ...
    'MarkerEdgeColor', [0.5, 0.1, 0.1], ...
    'LineWidth', 1.5);
xlabel('True Values', 'FontSize', 12);
ylabel('Predicted Values', 'FontSize', 12);
title('Training Set Fitting Performance', 'FontSize', 13, 'FontWeight', 'bold');
grid on

hold on
% Add fitting line
h1 = lsline;
set(h1, 'LineWidth', 2.5, 'LineStyle', '-', 'Color', [0.1, 0.4, 0.8]);
% Add diagonal line
plot([min(T_train), max(T_train)], [min(T_train), max(T_train)], ...
    'k--', 'LineWidth', 1.5);
hold off
legend('Training Data', 'Linear Fitting', 'y = x', 'Location', 'best');
set(gca, 'FontSize', 11, 'LineWidth', 1.2);

% Test set fitting plot
subplot(1,3,2)
scatter(T_test, T_sim2, 50, 'filled', ...
    'MarkerFaceColor', [0.4, 0.7, 0.4], ...
    'MarkerEdgeColor', [0.1, 0.5, 0.1], ...
    'LineWidth', 1.5);
xlabel('True Values', 'FontSize', 12);
ylabel('Predicted Values', 'FontSize', 12);
title('Test Set Fitting Performance', 'FontSize', 13, 'FontWeight', 'bold');
grid on
```

```
hold on
% Add fitting line
h2 = lsline;
set(h2, 'LineWidth', 2.5, 'LineStyle', '-', 'Color', [0.8, 0.3, 0.3]);
% Add diagonal line
plot([min(T_test), max(T_test)], [min(T_test), max(T_test)], ...
    'k--', 'LineWidth', 1.5);
hold off
legend('Test Data', 'Linear Fitting', 'y = x', 'Location', 'best');
set(gca, 'FontSize', 11, 'LineWidth', 1.2);

% All samples fitting plot
subplot(1,3,3)
all_pred = [T_sim1 T_sim2];
all_true = [T_train T_test];
scatter(all_true, all_pred, 40, 'filled', ...
    'MarkerFaceColor', [0.4, 0.6, 0.9], ...
    'MarkerEdgeColor', [0.1, 0.3, 0.6], ...
    'LineWidth', 1.5);
xlabel('True Values', 'FontSize', 12);
ylabel('Predicted Values', 'FontSize', 12);
title('All Samples Fitting Performance', 'FontSize', 13, 'FontWeight', 'bold');
grid on

hold on
% Add fitting line
h3 = lsline;
set(h3, 'LineWidth', 2.5, 'LineStyle', '-', 'Color', [0.9, 0.5, 0.2]);
% Add diagonal line
plot([min(all_true), max(all_true)], [min(all_true), max(all_true)], ...
    'k--', 'LineWidth', 1.5);
hold off
legend('All Data', 'Linear Fitting', 'y = x', 'Location', 'best');
set(gca, 'FontSize', 11, 'LineWidth', 1.2);

%% Calculate average metrics
R2_average = (R2_train + R2_test) / 2;
rmse_average = (rmse_train + rmse_test) / 2;

%% Create comprehensive evaluation metrics table - new addition
figure('Position', [100, 100, 800, 400])
% Prepare data
metrics = {'R^2'; 'RMSE'; 'MSE'; 'MAE'; 'MAPE'; 'MBE'; 'RPD'};
train_values = [R2_train; rmse_train; mse_train; MAE_train; MAPE_train; MBE_train; RPD_train];
```

```
cv_values = [R2_cv; rmse_cv; mse_cv; MAE_cv; MAPE_cv; MBE_cv; RPD_cv];
test_values = [R2_test; rmse_test; mse_test; MAE_test; MAPE_test; MBE_test; RPD_test];

% Create table display
uitable_data = [metrics, num2cell(train_values), num2cell(test_values), num2cell(cv_values)];
uitable_handle = uitable('Data', uitable_data, ...
    'ColumnName', {'Evaluation Metrics', 'Training Set', 'Test Set', '5-Fold CV'}, ...
    'RowName', [], ...
    'Position', [50, 50, 700, 300], ...
    'FontSize', 12, ...
    'ColumnWidth', {140, 130, 130, 130});

% Add title
annotation('textbox', [0.3, 0.9, 0.4, 0.05], ...
    'String', 'DBO-RBF Model Performance Evaluation Metrics', ...
    'FontSize', 14, 'FontWeight', 'bold', ...
    'HorizontalAlignment', 'center', ...
    'EdgeColor', 'none');

%% Print evaluation metrics - optimized version
disp('====================================================================')
disp('                   DBO-RBF Model Performance Evaluation Report')
disp('====================================================================')
fprintf('\n')

disp('----------------------- Training Set Error Metrics ----------------')
fprintf('Mean Absolute Error (MAE):         %10.4f\n', MAE_train)
fprintf('Mean Bias Error (MBE):             %10.4f\n', MBE_train)
fprintf('Mean Square Error (MSE):           %10.4f\n', mse_train)
fprintf('Root Mean Square Error (RMSE):     %10.4f\n', rmse_train)
fprintf('Coefficient of Determination (R²): %10.4f\n', R2_train)
fprintf('Residual Prediction Deviation (RPD): %8.4f\n', RPD_train)
fprintf('Mean Absolute Percentage Error (MAPE): %6.2f%%\n', MAPE_train*100)

disp('----------------------- Test Set Error Metrics --------------------')
fprintf('Mean Absolute Error (MAE):         %10.4f\n', MAE_test)
fprintf('Mean Bias Error (MBE):             %10.4f\n', MBE_test)
fprintf('Mean Square Error (MSE):           %10.4f\n', mse_test)
fprintf('Root Mean Square Error (RMSE):     %10.4f\n', rmse_test)
fprintf('Coefficient of Determination (R²): %10.4f\n', R2_test)
fprintf('Residual Prediction Deviation (RPD): %8.4f\n', RPD_test)
fprintf('Mean Absolute Percentage Error (MAPE): %6.2f%%\n', MAPE_test*100)

disp('----------------------- Five-Fold Cross-Validation ---------------')
```

```
fprintf('Pooled CV R²:                        %10.4f\n', R2_cv)
fprintf('Pooled CV RMSE:                      %10.4f\n', rmse_cv)
fprintf('Pooled CV MAE:                       %10.4f\n', MAE_cv)
fprintf('Pooled CV RPD:                       %10.4f\n', RPD_cv)
fprintf('Mean fold R²:                        %10.4f\n', mean(fold_R2))
fprintf('Mean fold RMSE:                      %10.4f\n', mean(fold_RMSE))

disp('----------------------- Comprehensive Evaluation ------------------')
fprintf('Average R²:                        %10.4f\n', R2_average)
fprintf('Average RMSE:                      %10.4f\n', rmse_average)

disp('====================================================================')
fprintf('\n')
```

Table S5 Detailed settings and implementation framework of the comparative regression algorithms.

Algorithm	Full name	Main role	Key settings used for comparison
DBO-RBF	Dung Beetle Optimizer-radial basis function regression	Optimized nonlinear RBF workflow	Population size = 30; maximum iteration = 100; fitness = training RMSE; optimized centers, widths and output weights
KNN	K-nearest neighbors regression	Distance-based local baseline	n_neighbors = 5; weights = distance; metric = Minkowski
Extra Trees	Extremely Randomized Trees regression	Highly randomized tree ensemble	n_estimators = 300; max_features = sqrt; random_state = 42
Random Forest	Random Forest regression	Bootstrap tree ensemble baseline	n_estimators = 300; max_features = sqrt; random_state = 42
SVR-RBF	Support vector regression with radial basis function kernel	Kernel-based nonlinear baseline	kernel = rbf; C = 10; gamma = scale; epsilon = 0.1
XGBoost	eXtreme Gradient Boosting regression	Gradient-boosted tree baseline	n_estimators = 300; learning_rate = 0.05; max_depth = 3; subsample = 0.8; colsample_bytree = 0.8
Standard RBF	Standard radial basis function regression	Unoptimized RBF reference	Centers initialized empirically; Gaussian widths estimated from center distances; linear output fitted by ridge regression
Ridge	Ridge regression	Regularized linear baseline	alpha = 1.0
MLP	Multilayer Perceptron regression	Neural-network nonlinear baseline	hidden_layer_sizes = (32, 16); activation = relu; solver = adam; alpha = 0.001; max_iter = 2000
PLSR	Partial Least Squares Regression	Chemometric linear latent-variable baseline	n_components selected within training data and limited by feature number and training sample size

Python implementation used for the comparative regression algorithms.

```
import numpy as np
from sklearn.model_selection import train_test_split, KFold, cross_val_predict
from sklearn.pipeline import Pipeline
from sklearn.preprocessing import StandardScaler
from sklearn.metrics import r2_score, mean_squared_error, mean_absolute_error
```

```
from sklearn.neighbors import KNeighborsRegressor
from sklearn.ensemble import ExtraTreesRegressor, RandomForestRegressor
from sklearn.svm import SVR
from sklearn.linear_model import Ridge
from sklearn.neural_network import MLPRegressor
from sklearn.cross_decomposition import PLSRegression
from xgboost import XGBRegressor

RANDOM_STATE = 42
FEATURE_COLUMNS = color_proxy_570_593 + texture_angle_0_40 + texture_angle_63_140
TARGETS = ['Cu', 'Fe', 'Mn', 'Zn']

models = {
    'KNN': KNeighborsRegressor(n_neighbors=5, weights='distance', metric='minkowski'),
    'Extra Trees': ExtraTreesRegressor(n_estimators=300, max_features='sqrt', random_state=RANDOM_STATE),
    'Random Forest': RandomForestRegressor(n_estimators=300, max_features='sqrt', random_state=RANDOM_STATE),
    'SVR-RBF': SVR(kernel='rbf', C=10, gamma='scale', epsilon=0.1),
    'XGBoost': XGBRegressor(n_estimators=300, learning_rate=0.05, max_depth=3,
                            subsample=0.8, colsample_bytree=0.8, random_state=RANDOM_STATE),
    'Ridge': Ridge(alpha=1.0),
    'MLP': MLPRegressor(hidden_layer_sizes=(32, 16), activation='relu', solver='adam',
                        alpha=0.001, max_iter=2000, random_state=RANDOM_STATE),
    'PLSR': PLSRegression(n_components=n_pls_components)
}

for target in TARGETS:
    X = data[FEATURE_COLUMNS].values
    y = data[target].values
    X_train, X_test, y_train, y_test = train_test_split(
        X, y, test_size=0.30, random_state=RANDOM_STATE
    )
    for name, estimator in models.items():
        model = Pipeline([('scaler', StandardScaler()), ('regressor', estimator)])
        model.fit(X_train, y_train)
```

```
y_train_pred = model.predict(X_train)
y_test_pred = model.predict(X_test)
y_full_pred = model.predict(X)
kf = KFold(n_splits=5, shuffle=True, random_state=RANDOM_STATE)
y_cv_pred = cross_val_predict(model, X, y, cv=kf)
metrics = {
    'train_R2': r2_score(y_train, y_train_pred),
    'test_R2': r2_score(y_test, y_test_pred),
    'full_R2': r2_score(y, y_full_pred),
    'cv_R2': r2_score(y, y_cv_pred),
    'test_RMSE': mean_squared_error(y_test, y_test_pred, squared=False),
    'test_MAE': mean_absolute_error(y_test, y_test_pred)
}
```
